# Spatio‐Temporal Plasticity of Root Exudation in Three Temperate Tree Species: Effects of Season, Site, and Soil Characteristics

**DOI:** 10.1111/ppl.70681

**Published:** 2025-12-17

**Authors:** Melissa Wannenmacher, Simon Haberstroh, Jürgen Kreuzwieser, Trung Hieu Doan, Jörg Niederberger, Jörg Prietzel, Friederike Lang, Christiane Werner

**Affiliations:** ^1^ Ecosystem Physiology, Faculty of Environment and Natural Resources University of Freiburg Freiburg im Breisgau Germany; ^2^ Silviculture, Faculty of Environment and Natural Resources University of Freiburg Freiburg im Breisgau Germany; ^3^ Soil Ecology, Faculty of Environment and Natural Resources University of Freiburg Freiburg im Breisgau Germany; ^4^ Renaturation Ecology, TUM School of Life Sciences Technical University of Munich Munich Germany

**Keywords:** *Acer pseudoplatanus*, *Fagus sylvatica*, *Picea abies*, plant–soil interaction, root exudation

## Abstract

Root exudation provides a constant carbon input to the rhizosphere and is therefore a very important factor in shaping this hotspot of biological activity. Nonetheless, data on root exudation and its spatio‐temporal plasticity are scarce. This study provides insights into compound‐specific root exudation in three temperate tree species in two seasons (late spring and late summer) and two soil compartments (forest floor and the top mineral soil), including the effect of soil chemistry. At four sites with differing mean annual temperatures and soil phosphorus levels, root exudates were sampled using an in situ cuvette‐based system and analysed by gas chromatography–mass spectrometry. We found seasonally and spatially varying site‐ and species‐specific exudation patterns. While the seasonal pattern was similar among species and sites, with higher exudation rates in late spring, soil compartment‐specific exudation depended on species and site. *Acer pseudoplatanus* tended to exude more into the mineral soil at warmer sites, while *Picea abies* exuded more in the mineral soil at all sites. Exudation by *Fagus sylvatica* was independent of the soil compartment. A significant correlation between exchangeable soil cations and specific compounds exuded by *F. sylvatica* and *P. abies* was found*.* Exudation of specific compounds in *F. sylvatica* increased with the concentration of exchangeable Mg, Al, and Fe, whereas exudation rates in *P. abies* decreased with most base cations' concentration, while sugar exudation increased with the exchangeable non‐base cations Al and Fe. These results demonstrate that root exudation is dynamically adjusted to the species‐specific nutritional needs governed by site, season, and soil characteristics.

## 1 Introduction

Root exudates play a major role in carbon and nutrient cycling of forests and transform the rhizosphere into a hub of biological activity. Various definitions of the term root exudation can be found, which comprise different selections of compounds. Most commonly, it includes organic compounds released by the living plant root, which distinguishes it from rhizodeposition more commonly seen as compounds set free by dead plant roots (Massalha et al. 2017). In this study, we use the term root exudates for soluble chemical compounds, which are released by living plant roots. Numerous functions of root exudates have been identified, including defence against pathogens, attraction of beneficial microbes, and plant–plant communication (Dannenmann et al. 2009; Huang et al. 2014; Rohrbacher and St‐Arnaud 2016). Through the so‐called priming effect, plants make organically bound nutrients bioavailable by providing easily degradable carbon (C) compounds to microorganisms as an energy source to decompose more recalcitrant components in the soil (Rohrbacher and St‐Arnaud 2016; Tückmantel et al. 2017). Ruf et al. (2006) state that root exudates are, next to litter and soil organic matter (SOM), one of the three primary C sources in the soil food web, which even reach the third trophic level, such as predatory mites. Moreover, rhizodeposition, even in small amounts, is responsible for up to one third of C— and nitrogen (N)— mineralisation in temperate forest soils (Finzi et al. 2015).

Next to the priming effect, plants are able to exude compounds, which directly increase the mobility and bioavailability of nutrients (Rohrbacher and St‐Arnaud 2016), demonstrating that nutrient uptake and root exudation are closely interrelated. However, there is no clear pattern on how nutrient availability influences root exudation and how root exudation rate and quality mediate nutrient uptake (Williams et al. 2022). While generally decreased exudation under phosphorus (P) limiting conditions has been reasoned by decreased meristematic activity by Canarini et al. (2019), other studies found increased exudation of organic acids when P was limiting (Jones et al. 2009; Vives‐Peris et al. 2020). Supporting this, increased exudation of organic acids under Fe limiting conditions was found by Vives‐Peris et al. (2020), coupled with an increase of exudation of sugars (Vives‐Peris et al. 2020) and phenolics (Vranova et al. 2013). A decrease and increase of 2‐benzopyranones exudation under P and Fe limiting conditions, respectively, has also been observed (Pantigoso et al. 2021). Contrasting those findings, a recent study on beech suggests that there is no effect of P availability on root exudation (Leuschner et al. 2022). However, this latter study examined the total exuded C not looking at organic acids specifically. Hence, the increased organic acid exudation under P limiting conditions might be compensated by a reduction of exudation in other compound groups. Exchangeable aluminium (Al^3+^) abundance also seems to increase the exudation of organic acids (Jones et al. 2009; Canarini et al. 2019). Hereby, malate, pyruvate, and citrate have been identified to help withstand Al toxicity (Pantigoso et al. 2021). The effect of other nutrients on root exudation is less studied. Nonetheless, it has been suggested that deficits of boron (B), zinc (Zn), and manganese (Mn) lead to increased exudation due to a loss of membrane functioning (Vranova et al. 2013). Further, potassium (K) starvation has been found to inhibit the exudation of sugars (Vives‐Peris et al. 2020). Next to the nutrient composition, more general site conditions also impose an effect on root exudation. In beech, a tripling of root exudation C rate was observed when the acidity doubled (Meier et al. 2020). Another study on *Lactuca sativa* did not find such a stringent relationship with pH but identified the soil type as an important factor influencing root exudation (Neumann et al. 2014). Salt stress, for example, also led to higher exudation of specific compounds in Arabidopsis and walnut (Vives‐Peris et al. 2020; Pantigoso et al. 2021). Most of the above‐mentioned studies were conducted on crops and grasses. Despite their importance, only a little is known about root exudation rates, composition, and strategies in different temperate tree species across seasons and soil compartments (Ritter et al. 2025). The fewer studies on trees might be due to the more challenging sampling procedure, especially when working in forests. Compared to grasses, trees use a relatively smaller share of photoassimilates for root exudates (Vives‐Peris et al. 2020), which seems to vary with species and site condition, ranging from 0.6% in beech (Brunn et al. 2022) to 5% in loblolly pine (Phillips et al. 2011) according to previous studies. Nonetheless, root exudation of trees is an important factor for the forest environment.

Root exudates, for instance, play a major role in shaping the composition and thickness of the forest floor (Prescott and Grayston 2013; Finzi et al. 2015). In the present work, the forest floor is defined as the uppermost layer of the forest soil, which mainly consists of litter in various stages of decomposition or transformation with a minimum of 15% organic C in mass (Wachendorf et al. 2023) and therefore represents a stock of organically bound nutrients. The forest floor has been identified as an important nutrient source for forest trees, especially at nutrient‐poor sites (Lang et al. 2017). In line with this, indications that the presence of organic N, as present in the forest floor, increased exudation have been found (Rohrbacher and St‐Arnaud 2016; Tückmantel et al. 2017; Vives‐Peris et al. 2020). Tückmantel et al. (2017) found that ectomycorrhiza (EM) associated tree species exuded more C compared to arbuscular mycorrhiza (AM) associated species. EM‐tree associations are able to produce extracellular enzymes and follow an organic nutrient economy, while AM‐tree associations often lack this feature and follow an inorganic nutrient economy (Phillips et al. 2013; DeForest and Snell 2020). The inorganic nutrient economy is characterized by a rapid turnover of the AM plant litter with a comparatively high quality. In contrast, EM plant litter has a lower quality, which leads to a slower turnover and therefore a larger nutrient storage pool in the organic layer. Hence, in theory, EM‐associated tree species should show a higher specialization on nutrient uptake from organic soil material relying on a tighter nutrient recycling in the forest compared to AM‐associated tree species, which rather acquire nutrients from mineral sources. Since different tree species follow different nutrient uptake strategies, it is likely that they also follow different root exudation strategies. The source of nutrients in forest floors is mainly organic matter, while in the mineral soil, inorganic sources play a bigger role, which could be mirrored in the quality and quantity of root exudates in the different tree species (Meier et al. 2020).

A seasonally varying nutrient demand could also be reflected in seasonal exudation patterns. Strong growth in spring increases the plant's nutrient demand, while during the period before dormancy in late summer and autumn a smaller amount is needed (Carrara et al. 2004). At the same time, photosynthetic activity is higher in spring, allowing for C investment in root exudates. However, Tückmantel et al. (2017) did not find variation in exudation across seasons (within one growing season) in beech. Also, the variation of exudation within days or weeks seems to be low (Phillips et al. 2008). On the other side, there are studies that found seasonal exudation patterns (Jakoby et al. 2020; Chen et al. 2023). Here, the predominant environmental conditions might confound seasonal impacts. For instance, water availability has been shown to play a decisive, yet contradictory role: while some studies found drought, that is, reduced soil water content, to cause higher exudation rates (Preece et al. 2018; Meier et al. 2020; Brunn et al. 2022; Leuschner et al. 2022), others found the opposite effect (Dannenmann et al. 2009; Li et al. 2025). Gargallo‐Garriga et al. (2018) suggest that exudation is increased under mild water stress, where, for instance, mucilage ameliorates soil penetration. However, under severe drought, exudation is reduced to save water within the plant (Gargallo‐Garriga et al. 2018). These contradictory findings reflect the lack of knowledge in this field.

The main objectives of this study were to investigate (i) the seasonal exudation patterns of the two EM‐associated tree species European beech ( *Fagus sylvatica* ) and Norway spruce ( *Picea abies* ), and the AM‐associated tree species Sycamore maple ( *Acer pseudoplatanus* ), then investigate (ii) exudation patterns in the forest floor and the upper 5 cm of the mineral soil at four temperate forest sites with differing abiotic conditions. Finally, we aimed at (iii) investigating the influence of soil chemistry on exudation patterns of the three species.

We hypothesize that trees can adjust their exudation (quantity and composition) on a temporal and a small spatial scale, such as between soil compartments, to their species‐specific nutritional needs in dependence on the distribution of nutrient availability in the soil. We hereby expect the EM‐associated species (beech and spruce) to show a generally higher exudation, which is more dominant in the forest floor, compared to a generally lower and mineral soil‐focused exudation in the AM‐associated species (maple).

## 2 Material and Methods

### 2.1 Study Sites

**FIGURE 1:**
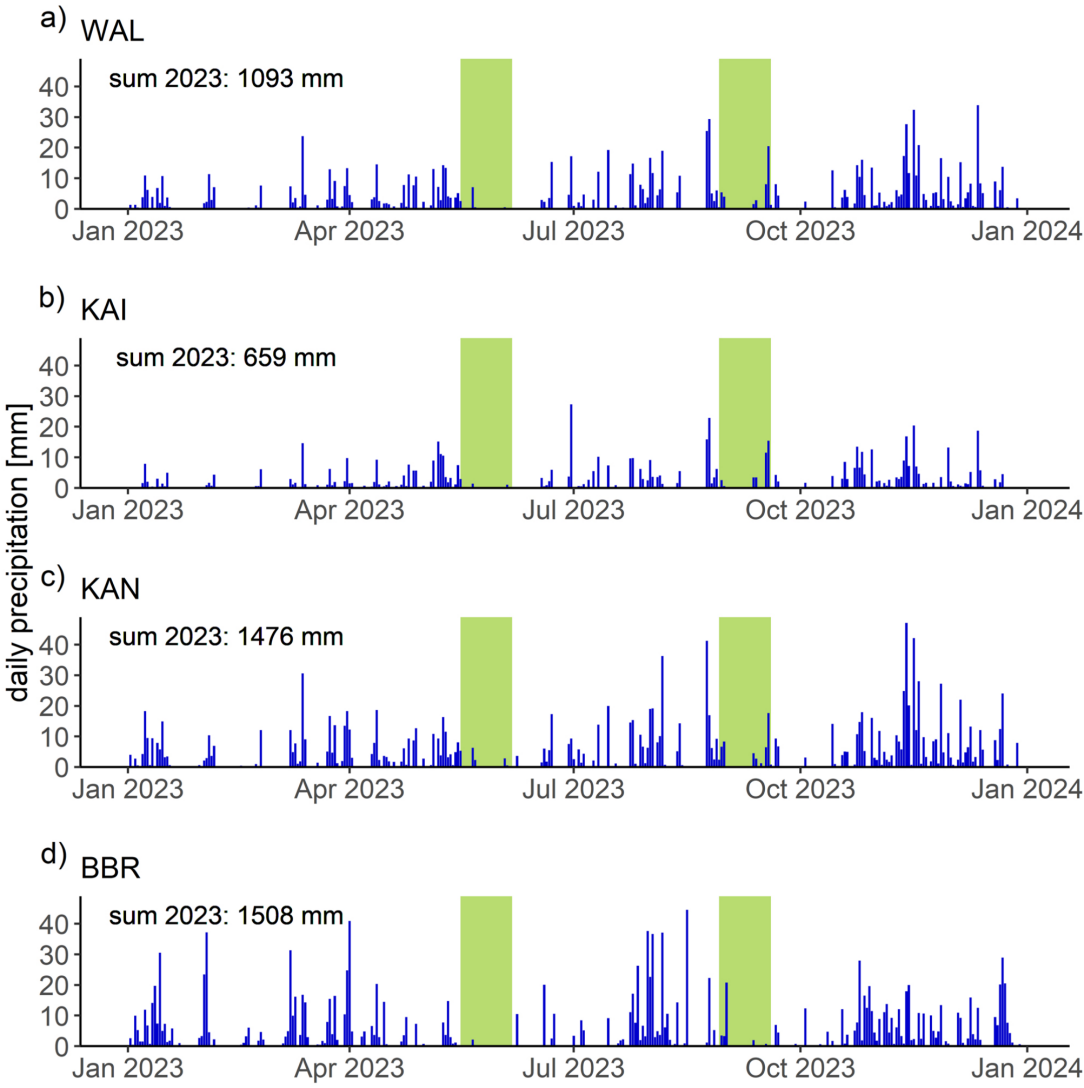
Precipitation pattern at the study sites in the sampling year. Daily precipitation data of the year 2023 from the DWD 1 × 1 km grids (DWD 2025b) for the sites WAL in (a), KAI in (b), KAN in (c) and BBR in (d). Sampling periods are colored in light green.

Samples were taken at four European beech ( *Fagus sylvatica* )‐dominated temperate forest sites. Waldkirch (WAL) and Kandel (KAN) are located in the Black Forest in southwest Germany. The site Kaiserstuhl (KAI) is situated in the same region, but in the upper Rhine valley. Bad Brückenau (BBR) lies in the Rhön mountains in Central Germany. Basic information on the sites can be found in Table 1. The sites mainly differ in mean annual temperature (MAT) and phosphorus (P) level in the soil (Tables 1 and 2). The two sites at lower altitudes, WAL and KAI, showed a higher MAT in the reference period 1991–2020 than the sites KAN and BBR at higher altitudes. Being located on basalt rock, KAI and BBR can be classified as P‐rich, while the other two sites located on gneiss rock showed significantly lower P stocks in the forest floor and the mineral soil and can be considered as P‐poor (Table 2). The sites with higher MAT, KAI and WAL, are less acidic than the cooler sites. The soil type was classified according to WRB 4th edition (IUSS Working Group WRB 2022) and the humus form according to the current German soil classification system (KA6; Wachendorf et al. 2023). Long‐term mean annual precipitation was lowest at KAI with 775 mm yr.^−1^ and highest at KAN with 1457 mm yr.^−1^ in the reference period from 1991 to 2020. Temperature and precipitation data were deducted from 1 × 1 km grid data HYRAS‐DE‐TAS (for MAT) and HYRAS‐DE‐PR (for MAP) provided by the DWD Climate Data Center (CDC; DWD 2025a, 2025b). Additionally, for 2023, daily precipitation data in the same 1 × 1 km grids were extracted from HYRAS‐DE‐PR (v6.1; DWD 2025b) to describe general precipitation patterns. The annual precipitation patterns were similar at all sites in 2023: after a period of low precipitation in May/June 2023, frequent summer rainfall occurred, followed by an autumn with rather low rainfall. Both our sampling campaigns in late spring and late summer fell into these periods of rather low precipitation (Figure 1). However, there were regular rainfall events in the 6 weeks before the sampling periods at all sites (Figure 1). Next to precipitation patterns, atmospheric conditions also impact the water status of ecosystems. To gain a more holistic picture of drought conditions at the sites, we retrieved the Standardised Precipitation Evaporation Index (SPEI; Vicente‐Serrano et al. 2010) for the four sites from the SPEI database (https://spei.csic.es/spei_database/; Beguería et al. 2010; Vicente‐Serrano et al. 2010). The values calculated on the basis of the six previous months were chosen here.

**TABLE 1:** Site information with coordinates, altitude, mean annual temperature (MAT), mean annual precipitation (MAP) from the years 1991–2020 (DWD, 2025), parent material, soil type, humus form, tree species composition (based on basal area of all tree stems, DBH ≥ 7 cm), number of trees per hectare, and basal area per hectare.

	WAL	KAI	KAN	BBR
Coordinates	48.1° N, 7.9° E	48.1° N, 7.7° E	48.1° N, 8.0° E	50.4° N, 9.9° E
Altitude (m.a.s.l.)	415	396	1163	809
Parent material	Gneiss	Basalt	Gneiss	Basalt
Soil type	Dystric Cambisol	Skeletic Umbrisol	Dystric Skeletic Rhodic Cambisol	Eutric Cambisol
Humus form	Typical F‐mull	Typical F‐mull—typical moder	Typical moder	Typical F‐mull—typical moder
MAT (°C)	10.0	10.3	6.0	6.7
MAP (mm)	1082	775	1457	1090
Tree species composition (%)	Maple (0.1), beech (34), spruce (8), other (58)	Maple (1), beech (96), other (3)	Maple (0),^a^ beech (51), spruce (46), other (3)	Maple (2), beech (98), spruce (0)^a^
Number of trees (ha^−1^)	294	272	466	350
Basal area (m^2^ ha^−1^)	43	26	33	40

^a^ Sampling took place outside but in close proximity to the designated study area due to a lack of individuals of the respective species in the study plot.

**TABLE 2:** Exchangeable cation concentrations, cation exchange capacity (CEC), base saturation (BS), and pH (H_2_O) ± standard error at the four study sites Waldkirch (WAL), Kaiserstuhl (KAI), Kandel (KAN), and Bad Brückenau (BBR), in the forest floor and mineral soil.

	WAL		KAI		KAN		BBR	
	Forest floor	Mineral soil	Forest floor	Mineral soil	Forest floor	Mineral soil	Forest floor	Mineral soil
P (mg g^−1^)	0.7 ± 0.0	0.5 ± 0.0	1.2 ± 0.1	1.4 ± 0.1	0.9 ± 0.1	0.9 ± 0.0	1.3 ± 0.2	2.1 ± 0.1
Al^3+^ (μmol_c_ g^−1^)	0.8 ± 0.2	8.7 ± 2.0	0.6 ± 0.3	8.9 ± 6.6	21.0 ± 11.5	77.2 ± 12.7	12.7 ± 6.1	49.6 ± 3.7
Ca^2+^ (μmol_c_ g^−1^)	225.4 ± 27.3	28.3 ± 5.4	304.6 ± 22.6	100.1 ± 44.6	207.3 ± 57.9	16.7 ± 8.1	167.5 ± 28.3	48.2 ± 7.5
Fe^2+^ (μmol_c_ g^−1^)	0.1 ± 0.0	0.1 ± 0.0	0.1 ± 0.0	0.8 ± 0.7	2.3 ± 1.1	6.5 ± 1.5	0.9 ± 0.4	2.3 ± 0.4
K^+^ (μmol_c_ g^−1^)	55.8 ± 1.8	4.4 ± 0.6	39.8 ± 5.8	4.7 ± 0.8	21.3 ± 3.0	4.4 ± 0.5	19.7 ± 2.9	6.9 ± 1.1
Mg^2+^ (μmol_c_ g^−1^)	51.3 ± 8.9	5.4 ± 0.9	101.7 ± 4.2	21.7 ± 3.4	69.9 ± 11.9	10.6 ± 1.9	88.4 ± 16.1	25.3 ± 4.5
Mn^2+^ (μmol_c_ g^−1^)	52.3 ± 8.8	16.9 ± 2.2	14.9 ± 2.7	2.4 ± 0.6	15.1 ± 4.6	1.3 ± 0.7	15.9 ± 1.7	6.7 ± 0.4
Na^2+^ (μmol_c_ g^−1^)	2.5 ± 0.6	0.3 ± 0.1	2.0 ± 0.4	0.9 ± 0.2	1.3 ± 0.2	0.7 ± 0.1	2.2 ± 0.6	1.2 ± 0.3
CEC (μmol_c_ g^−1^)	389.8 ± 23.2	67.9 ± 5.7	465.2 ± 30.4	142.6 ± 43.2	370.9 ± 56.9	138.1 ± 8.2	317.1 ± 42.2	151.7 ± 12.5
BS	85.7 ± 3.6	55.3 ± 6.3	96.2 ± 0.8	83.2 ± 11.2	74.0 ± 10.1	25.8 ± 9.3	83.8 ± 4.9	51.6 ± 4.8
pH (H_2_O)	5.9 ± 0.1	5.1 ± 0.0	6.3 ± 0.2	5.5 ± 0.5	4.4 ± 0.2	4.0 ± 0.1	5.1 ± 0.2	4.5 ± 0.0

Stand characteristics of the study areas were quantified based on a stand inventory campaign conducted in summer 2023 (Table 1). In each study area, species identity and diameter at breast height (DBH, measured at 1.3 m above ground) were recorded for all tree stems with DBH ≥ 7 cm. Stand basal area, expressed in square meters per hectare (m^2^ ha^−1^), was calculated as the sum of the cross‐sectional areas at breast height of all recorded stems, divided by the inventory area. Tree species composition at the stand level was determined as the proportion of each species' basal area relative to the total stand basal area. Beech had the highest share of basal area across all sites, ranging from 34% at WAL to 98% at BBR. Spruce and maple combined had a low basal area share, varying between 1% and 8% across sites. Stand basal area ranged from 26 m^2^ ha^−1^ at KAI to 43 m^2^ ha^−1^ at WAL. Tree density was highest at KAN with 466 stems per hectare, and lowest at KAI with 272 stems per hectare (Table 1).

Soil and forest floor pH (H_2_O) of air‐dried samples (40°C) was determined using double deionized water at a ratio of 1:5 (soil: solution). Samples were analysed using the Metrohm CH/789 Robotic Sample Processor XL, measuring chain: Micro El.Cone 16 WOC.

Exchangeable cations were extracted from the soil using NH_4_Cl at a concentration of 0.5 mol l^−1^. The extracted cations (Ca, Mg, K, Na, Mn, Al, and Fe) were quantified by inductively coupled plasma optical emission spectroscopy (IPC‐OES 5800, Agilent) and referenced to the soil mass. The proton concentration (used for CEC calculation) was determined by titration using 0.05 mol NaOH and with the Metrohm CH, Titrando 905/789 Robotic Sample Processor XL, measuring chain: Micro El.Cone 16 WOC.

For the total soil element content analysis, the dried and milled soil and forest floor samples underwent HNO_3_ microwave pressure digestion at 170°C, followed by inductively coupled plasma atomic emission spectroscopy (ICP‐OES 5800, Agilent).

Next to the differences in P stocks at the sites, the cooler sites BBR and KAN showed significantly higher concentrations of exchangeable non‐base cations Al^3+^ and Fe^2+^ compared to KAI and WAL in the forest floor (*p* = 0.03) and the mineral soil (*p* < 0.01), while for the exchangeable non‐base cation Mn^2+^, the concentration was significantly highest in the mineral soil at WAL compared to the other sites (*p* < 0.01). Site differences in exchangeable base cation concentration were less pronounced; however, the Mg^2+^ concentrations in the mineral soil were significantly higher at the P‐rich sites BBR and KAI compared to the P‐poor sites (*p* < 0.01). A summary of soil element concentrations, pH, cation exchange capacity (CEC), and the base saturation (BS) in the soil compartments relevant for exudation sampling can be found in Table 2. The given values were averaged over three to five sampling points per site and soil compartment. The forest floor thickness was measured and averaged over three spots around each sampling tree with a distance of 1.5 m from the stem. Small holes were dug to obtain a flat vertical surface, where the forest floor—mineral soil interface was clearly visible. The forest floor thickness ranged from a minimum of 0.2 cm at WAL (late spring: 2.6 ± 2.0 cm (mean ± standard deviation), late summer: 2.1 ± 1.6 cm) over KAI (late spring: 2.1 ± 1.0 cm, late summer: 2.0 ± 1.8 cm) and BBR (late spring: 6.1 ± 1.7 cm, late summer: 4.2 ± 2.0 cm) to a maximum of 10.0 cm at KAN (late spring: 6.3 ± 1.7 cm, late summer: 3.9 ± 2.0 cm).

### 2.2 Sampling Design

Sampling took place on five trees per species per site in two campaigns in late spring and late summer in 2023. No spruce trees were present in the designated study areas at KAI and BBR, and no maple at KAN. At BBR and KAN, trees of the missing species could be found in close proximity to the study area, where differences in environmental conditions can be expected to be minor. The DBH of the sampling trees ranged from 3.2 to 40.4 cm in maple, from 25.8 to 47.8 cm in beech, and from 31.8 to 63.3 cm in spruce (Table 3). The smaller DBH in maple was due to the absence of larger individuals of maple at the selected sites. The sampling campaigns lasted from the 16th of May to the 6th of June (KAI: 16/05–20/05, WAL: 22/05–26/05, KAN: 28/05–01/06, BBR: 03/06–06/06) and from the 29th of August to the 19th of September (KAN: 29/08–02/09, WAL: 04/09–07/09, BBR: 10/09–13/09, KAI: 16/09–19/09). In all campaigns, root exudates from the same trees were sampled to avoid sampling artefacts through specific exudation patterns of individual trees. In each campaign, two roots per tree were investigated, one in the forest floor and one in the mineral soil. Additionally, between 7 and 10 control cuvettes per site without roots were exposed to the same sampling procedure, as described below.

**TABLE 3:** Mean DBH ± standard deviation (in cm) of the sampling trees (five per site and species) at the four sites for the tree species *Acer pseudoplatanus* (maple), *Fagus sylvatica* (beech), and *Picea abies* (spruce).

	Maple	Beech	Spruce
WAL	13.9 ± 3.6	43.2 ± 4.0	44.8 ± 10.0
KAI	7.6 ± 5.3	36.5 ± 7.5	—
KAN	8.9 ± 3.7	33.5 ± 3.0	48.3 ± 6.7
BBR	20.7 ± 13.5	39.3 ± 7.1	38.5 ± 5.7

### 2.3 Root Exudate Collection

An in situ cuvette‐based approach based on Phillips et al. (2008) was used for the exudate collection. Two roots per tree, one in the forest floor and one in the mineral soil were excavated and traced back to the tree to correctly identify the tree individuum. Only undamaged fine roots with a diameter below 2 mm were used. Soil particles were carefully removed with water and tweezers while the roots were still attached to the tree. Tap water was used for cleaning instead of distilled water to avoid stressing the root through a high osmotic potential within and outside the plant cells. In case of visible damage through the cleaning process, another root branch was selected. Aluminium foil was wrapped around the cleaned root to let it recover from the excavation and cleaning process for 24 h. The aluminium foil was covered with litter to avoid animal attraction. After this recovery time, the root was briefly rinsed with tap water and then placed in a cuvette consisting of a 30 mL plastic syringe with Luer‐Lock fitting (B.Braun) with a 3‐way‐valve (Teqler) attached to it. The syringe plunger was removed and the outlet was covered with glass wool. 3 mm glass beads and 10 mL of a C‐free dilute nutrient solution (0.5 mM NH_4_NO_3_, 0.1 mM KH_2_PO_4_, 0.2 mM K_2_SO_4_, 0.15 mM MgSO_4_*7H_2_O, and 0.4 mM CaCl_2_*2H_2_O) were added to imitate the soil environment. Afterwards, the cuvette was sealed with parafilm to avoid contamination. To allow root exudation to stabilise in the cuvette environment, an equilibration period of 24 h followed. Thereafter, the nutrient solution was removed using a syringe and two flushes with a C‐ and N‐free dilute nutrient solution (0.1 mM KH_2_PO_4_, 0.2 mM K_2_SO_4_, 0.15 mM MgSO_4_*7H_2_O, and 0.4 mM CaCl_2_*2H_2_O) were performed before adding 5 mL of the same C‐ and N‐free solution as exudation capturing solution. After 24 h, the capturing solution was removed and two flushes of 10 mL were performed to capture the exudates attached to the glass beads and the root. The retrieved solution was immediately filtered through a 0.2 μm sterile syringe filter. In the field, exudation solutions were kept in a cooling box and covered by frozen ice pads before being frozen to −80°C at the lab until further analysis. The control cuvettes were handled in the exact same way as the cuvettes containing roots. The roots in the cuvettes were cut, cooled at ~8°C until scanning. The root surface area was determined using the software WinRHIZO 2021a 32‐Bit (Regent Instruments Inc.) with a scanner (Perfection V850 Pro, Seiko Epson Corporation).

### 2.4 Root Exudate Analysis With GC–MS

The frozen root exudate samples were lyophilised for ~90 h and the dried exudates were derivatised. Derivatisation was conducted as specified in Maurer et al. (2021). In brief, the lyophilised exudates were solved in 100 μL of a 20 mg mL^−1^ solution of methoxyamine hydrochloride in anhydrous pyridine. After centrifugation, 20 μL of the prepared solution were incubated on the thermoshaker at 30°C and 1400 rpm for 90 min. Then, 35 μL of N‐methyl‐N‐(trimethylsilyl)‐trifluoroacetamide (MSTFA) were added and samples were shaken at 37°C and 1400 rpm for 30 min. The derivatives were analysed by GC–MS (GC 7820 gas chromatograph coupled to a 5975C MSD, Agilent). Parameter settings were the same as specified in Kreuzwieser et al. (2009) with a split of 2:1. For peak detection, identification, and peak area determination, the MASSHUNTER Quantitative Analysis software (Agilent Technologies) with the Golm Metabolome Database (2011) was used. Only compounds with a match factor larger than 60 were included in the analysis. The identified compounds were classified into 11 groups based on their chemical properties: amino acids, organic acids, fatty acids (containing more than seven carbon atoms), inorganic acids, alcohols, sugars, N‐containing and N‐free aromatic compounds, other N‐containing compounds, other hydrocarbons, and others, which comprises non‐identified compounds.

### 2.5 Quantification of Exudation Rates

For all analysed compounds, the peak area means of the controls from one sampling period and site were subtracted from the peak areas of the respective samples to account for possible contamination. Exudation rates were calculated based on the amount of the exuded compound per the root surface area and per exposure time of roots in the cuvette (24 ± 1 h).

The following compounds were quantified using authentic standards: alanine, aspartic acid, boric acid, citric acid, cysteine, fructose, galactose, glutamic acid, glycerol, lactic acid, leucine, malic acid, N,N‐dimethyl‐glycine, phenylalanine, propane‐1,3‐diol, pyruvic acid, sucrose, triethanolamine, and uric acid. The peak areas of those compounds covered > 75% of the total peak area in the samples. For the compounds not covered by the mentioned standards, a mean quantification factor was calculated from the standards within the same group. A list of the identified compounds, their classification, and the standard used for quantification can be found in the Supporting Information (Tables S1 and S2).

Root exudation has been reported in different units, for example, μmol C g_dry root_^−1^ h^−1^ when focusing on plant physiological processes and g C (10 cm)^−1^ m^−2^ a^−1^ when focusing on ecosystem carbon fluxes (Tückmantel et al. 2017). In this study, we chose the root surface area as a reference (ng cm^−2^ h^−1^), since it is a better predictor for root exudation (Jakoby et al. 2020) and represents the direct exchange layer between the root and the rhizosphere, where root exudates and nutrients are transferred. Furthermore, it allows for valid comparisons between species.

### 2.6 Statistical Analysis

All data were analysed using R (version 4.3.2, R Core Team 2024) in RStudio (version 2024.12.0). The Kruskal–Wallis test combined with the post hoc Dunn test was used to compare soil elemental concentrations represented in Table 2 between sites. To determine significant differences in exudation between seasons and soil compartments, the Wilcoxon test was used since the normality of the residuals of the exudation data set was not given. The effect of season/soil compartment was first tested soil compartment/season‐specific. Since the seasonal pattern proved to be similar in both soil compartments, values were averaged over soil compartments. The strength of correlations between compound groups in the exudation and the concentration of soil elements and DBH was determined with the Spearman's rank correlation coefficient. Hereby, the soil compartment‐specific values of soil elements and exudation were used. Since the correlations between soil element concentrations and root exudation rates were similar for the forest floor and the mineral soil, the data were combined (Figure 4). Graphs for soil compartment specific regressions can be found in the Supporting Information (Figures S2–S10). To test for significances in those correlations, the function cor.test with the option ‘method = “spearman”’ was used. Regressions between compound groups in the exudation and the concentration of soil elements were visualised using a generalised linear smoothing.

## 3 Results

In total, 72 compounds detected by GC–MS were included in the analysis. The most abundant compounds were N,N‐dimethylglycine, triethanolamine, boric acid, and octadecadienoic acid, which covered ~60% of the exudation mass included in the analysis. The most important compound groups were amino acids, other N‐containing compounds, and inorganic acids. Sugars and N‐containing aromatic compounds were the least abundant compound groups; however, those groups showed a high responsiveness to environmental conditions. A full list of identified compounds, including the compound class, can be found in the Supporting Information (Tables S1 and S2).

**FIGURE 2:**
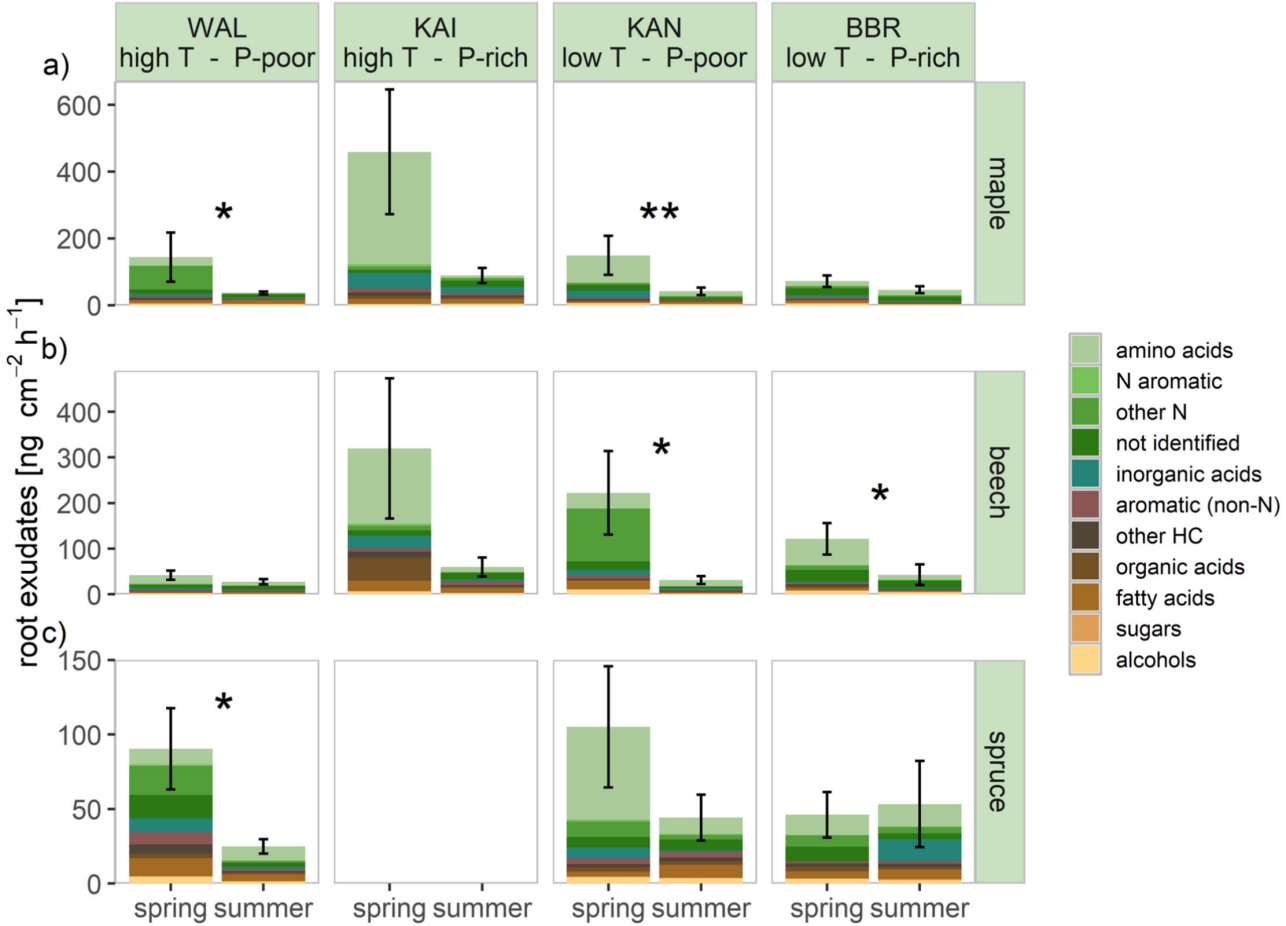
Seasonal variation in root exudation. Root exudation in late spring (spring) and late summer (summer) for maple in (a), beech in (b), spruce in (c) at the four study sites with different temperature (T) and soil phosphorus level (P). Colours code compound groups within the root exudates. Asterisks indicate significant differences (Wilcoxon signed‐rank test) between late spring and late summer (*p* ≤ 0.01: **, *p* ≤ 0.05: *). Note the different scales for different species in (a)–(c).

Amongst the tree species, maple and beech showed the highest exudation rates with 131 ± 30 ng cm^−2^ h^−1^ and 103 ± 23 ng cm^−2^ h^−1^ (mean across all samples and both seasons ± standard error), respectively, while spruce exuded significantly less than maple (*p* ≤ 0.01) with 61 ± 11 ng cm^−2^ h^−1^ (Figure 2). Amino acids made up the highest share of exudation with 48%, 37%, and 34% for maple, beech, and spruce, respectively. The share of all N‐containing compounds in the exudation of maple and beech made up more than one half with 60% and 54%, respectively, while for spruce they amounted to 47% of exudation. In contrast, spruce showed a higher share of non‐N‐hydrocarbons, such as aromatics, fatty acids, and sugars compared to the other species (Figure 2).

The difference in DBH between tree individuals did not prove to influence root exudation rates significantly (*p* > 0.05) with Spearman's rank correlation coefficients ρ = −0.11 (*p* = 0.64) in maple, *ρ* = 0.02 (*p* = 0.83) in beech, and *ρ* = −0.05 (*p* = 0.69) in spruce (Figure S11).

### 3.1 Seasonal Variation

The sum of exudation in late spring was, in general, higher in all species across all sites than in late summer, except for spruce at BBR, where exudation was (non‐significantly) higher in late summer (Figure 2). All tree species exuded more in late spring compared to late summer at all sites, with the exception of spruce at the low‐temperature P‐rich site BBR (Figure 2). The share of N‐containing compounds also decreased from late spring to late summer. In contrast, non‐N‐containing hydrocarbons, especially fatty acids and aromatics, were exuded at higher rates in late summer compared to late spring in all species.

### 3.2 Exudation in the Forest Floor and Mineral Soil

**FIGURE 3:**
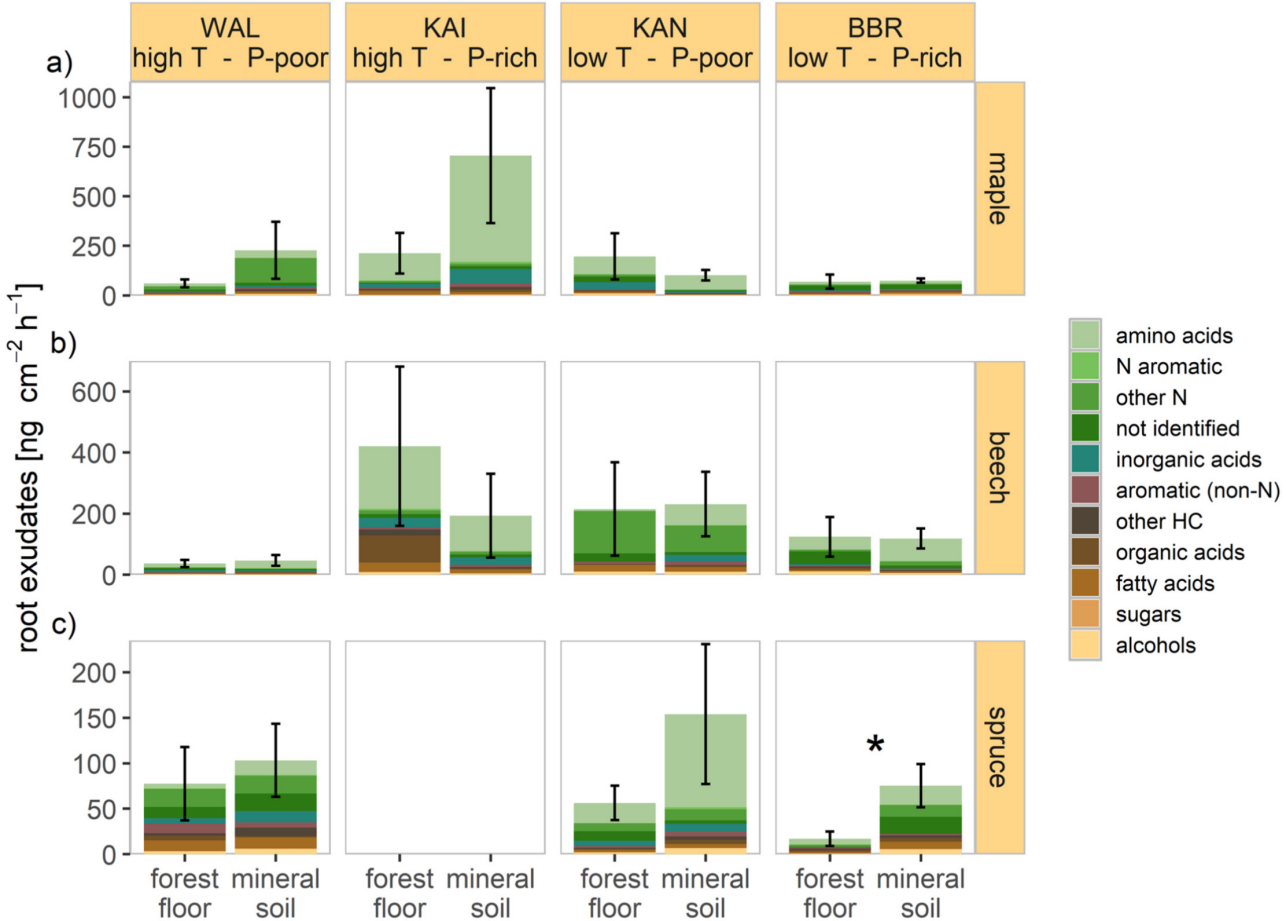
Root exudation in the forest floor and the mineral soil. Root exudation in late spring in the forest floor and the mineral soil for maple in (a), beech in (b) and spruce in (c) at the four study sites. Colours code compound groups within the exudates. Asterisks indicate significant differences between soil compartments (*p* ≤ 0.05). Note the different scales for different species.

Higher exudation rates in the mineral soil than in the forest floor were found in spruce across all sites and in maple at the two warmer sites WAL and KAI in late spring (although only significant for spruce at BBR, *p* < 0.05; Figure 3). Contrarily, at KAN, the low‐temperature, P‐poor site with a comparatively thick forest floor, we observed a higher exudation in the forest floor in maple (not significant). Root exudation rates were similar in the forest floor and the mineral soil for maple at BBR and for beech at all sites except KAI (high temperature, P‐rich). The share of all N‐containing compounds in the mineral soil was higher (74% in maple to 56% in spruce) than in the forest floor (56% in maple to 43% in spruce), whereas the share of organic and fatty acids was higher in the forest floor compared to the mineral soil in all species. For instance, in beech, the share of organic acids in the forest floor was 12%, but only 2% of all exuded compounds were organic acids in the mineral soil. In late summer, similar exudation patterns for all species between forest floor and mineral soil were found, although differences were less pronounced (Figure S1).

### 3.3 Exchangeable Cations

**FIGURE 4:**
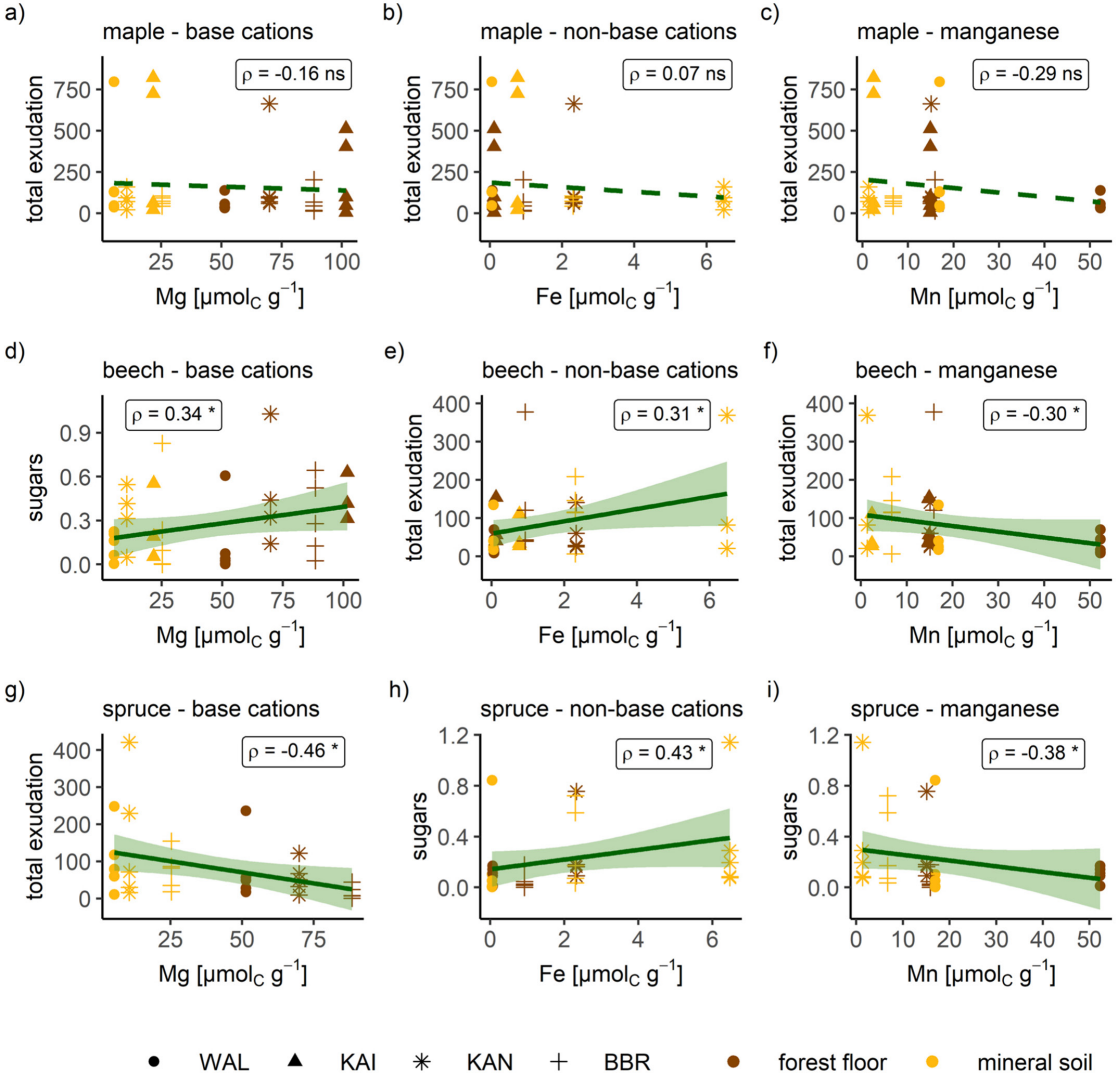
Root exudation under varying soil characteristics. Exudation (ng cm^−2^ h^−1^) in relation to exchangeable base cation concentrations in (a), (d) and (g), for exchangeable non‐base cation concentrations except Mn in (b), (e) and (h) and exchangeable Mn concentrations in (c), (f) and (i) in maple in (a)–(c), in beech in (d)–(f) and in spruce in (g)–(i). Shapes indicate the four different sites and the colors the soil compartment. *ρ* indicates the Spearman's rank correlation coefficient. Regression lines are illustrated by generalised linear smoothing and the shaded areas indicate the 95% confidence interval. Dashed regression lines are not significant. Note that variables and scales vary between panels.

Root exudates were significantly related to exchangeable soil cations (Tables 4, 5, 6). Hereby, exchangeable base cations (Ca^2+^, K^+^, Mg^2+^, and Na^+^) and exchangeable non‐base cations (Al^3+^, Fe^2+^ and Mn^2+^) led to distinct species‐ and compound‐specific responses (exemplarily illustrated in Figure 4). In general, Mn^2+^ concentration increased with decreasing concentrations of the other exchangeable non‐base cations, leading to inverse relationships to root exudates compared to Al^3+^ and Fe^2+^. The representation in Figure 4 combines data from the forest floor and the mineral soil, since the observed trends were similar for both compartments. Graphs with separate regression lines for the two compartments can be found in the Supporting Information (Figures S2–S10).

In maple, the total exudation tended to decrease with increasing exchangeable base cations and to increase with increasing non‐base cation concentrations (except Mn^2+^). However, those tendencies were not statistically significant, and some exudation compound groups showed inverse patterns (Table 4).

In beech, increasing exudation rates were observed for both increasing exchangeable base and non‐base cations (except Mn^2+^) across a wide range of exuded compound groups. Significant relationships could hereby be detected for Mg^2+^ and organic acids (Spearman's rank correlation coefficient *ρ* = 0.27), including fatty acids (*ρ* = 0.29), sugars (*ρ* = 0.34), and other compounds (*ρ* = 0.33) for base cations. With respect to exchangeable non‐base cations, Fe^2+^ and Mn^2+^ showed significant relationships with the total exudation rate (*ρ* = 0.31 for Fe^2+^, *ρ* = −0.30 for Mn^2+^), the exudation rate of other N compounds (*ρ* = 0.39 for Fe^2+^, *ρ* = −0.34 for Mn^2+^) and non‐N aromatic compounds (*ρ* = 0.37 for Fe^2+^, *ρ* = −0.41 for Mn^2+^) in beech. While an increase with Fe^2+^ concentration was observed, there was a decrease with increasing Mn^2+^ (Table 5).

In spruce, all exuded compounds decreased with increasing exchangeable base cations, while increasing non‐base cations (except Mn^2+^) tended to increase exudation rates, but not in all exuded compounds. Generally, under increasing base cation concentrations, spruce exuded significantly smaller rates in total and of amino acids and alcohols (ρ ranging from −0.34 to −0.47). On the other hand, under increasing non‐base cation concentrations (except Mn^2+^), spruce showed a significantly higher exudation rate of sugars (*ρ* = 0.35 for Al^3+^, *ρ* = 0.43 for Fe^2+^, *ρ* = −0.38 for Mn^2+^; Table 6).

**TABLE 4:** Correlation matrix of Spearman's rank correlation coefficient *ρ* between compound groups in the exudates of maple in late spring and the concentration of exchangeable cations in the soil with base cations on the left and non‐base cations on the right side of the matrix.

maple—*ρ*	Ca^2+^	K^+^	Mg^2+^	Na^+^	Al^3+^	Fe^2+^	Mn^2+^
Total exudation	−0.17	−0.25	−0.16	−0.25	0.05	0.07	−0.29
Amino acids	−0.25	−0.26	−0.28	−0.30	0.13	0.08	−0.23
Aromatic with N	−0.24	−0.25	−0.31	−0.33	0.04	−0.02	−0.20
Other N compounds	0.16	0.17	0.18	0.14	−0.03	0.02	0.07
Other	−0.13	−0.08	−0.04	−0.07	0.27	0.23	−0.08
Inorganic acids	0.07	0.00	−0.02	−0.06	−0.14	−0.07	−0.12
Aromatic without N	−0.05	−0.15	−0.05	−0.11	−0.02	−0.01	−0.23
Other hydrocarbons	−0.22	−0.23	−0.31	−0.37*	−0.05	−0.15	−0.08
Organic acids	−0.13	−0.19	−0.10	−0.16	0.10	0.12	−0.24
Fatty acids	0.17	0.11	0.12	0.07	−0.27	−0.27	0.09
Sugars	−0.08	−0.04	0.14	0.15	0.41	0.40	−0.15
Alcohols	−0.07	−0.05	0.07	0.01	0.26	0.29	−0.16

*Note:* Negative correlations are highlighted in shades of blue, positive ones in shades of red. Significant correlations are written in bold and marked with * for *p* ≤ 0.05 and ** for *p* ≤ 0.01.

**TABLE 5:** Correlation matrix of Spearman's rank correlation coefficient *ρ* between compound groups in the exudates of beech in late spring and the concentration of exchangeable cations in the soil with base cations on the left and non‐base cations on the right side of the matrix.

Beech—ρ	Ca^2+^	K^+^	Mg^2+^	Na^+^	Al^3+^	Fe^2+^	Mn^2+^
Total exudation	0.06	−0.03	0.23	0.14	0.14	0.31*	−0.30*
Amino acids	−0.08	−0.07	0.03	0.03	0.11	0.07	−0.04
Aromatic with N	−0.03	−0.08	0.04	0.03	−0.02	0.01	−0.11
Other N compounds	−0.17	−0.19	−0.01	−0.04	0.34*	0.39*	−0.34*
Other	0.26	0.25	0.33*	0.26	−0.02	0.15	0.03
Inorganic acids	0.07	0.01	−0.01	0.03	−0.17	−0.12	−0.01
Aromatic without N	0.04	−0.07	0.23	0.10	0.18	0.37*	−0.41**
Other hydrocarbons	0.02	−0.08	0.26	0.20	0.15	0.26	−0.20
Organic acids	0.20	0.10	0.27*	0.25	−0.11	0.05	−0.05
Fatty acids	0.20	0.08	0.29*	0.18	−0.08	0.14	−0.14
Sugars	0.19	0.03	0.34*	0.19	−0.01	0.26	−0.26
Alcohols	0.06	0.04	0.22	0.16	0.19	0.33*	−0.17

*Note:* Negative correlations are highlighted in shades of blue, positive ones in shades of red. Significant correlations are written in bold and marked with * for *p* ≤ 0.05 and ** for *p* ≤ 0.01.

**TABLE 6:** Correlation matrix of Spearman's rank correlation coefficient *ρ* between compound groups in the exudates of spruce in late spring and the concentration of exchangeable cations in the soil with base cations on the left and non‐base cations on the right side of the matrix.

Spruce—*ρ*	Ca^2+^	K^+^	Mg^2+^	Na^+^	Al^3+^	Fe^2+^	Mn^2+^
Total exudation	−0.34*	−0.34*	−0.46*	−0.46*	0.13	0.05	−0.29
Amino acids	−0.43*	−0.43*	−0.47**	−0.47**	0.21	0.12	−0.34
Aromatic with N	−0.28	−0.28	−0.43*	−0.38*	−0.01	−0.08	−0.12
Other N compounds	−0.05	−0.05	−0.26	−0.22	−0.20	−0.26	0.09
Other	−0.08	−0.08	−0.29	−0.27	−0.08	−0.08	−0.06
Inorganic acids	−0.13	−0.13	−0.20	−0.20	−0.08	−0.06	0.00
Aromatic without N	−0.12	−0.12	−0.21	−0.17	0.05	0.09	−0.11
Other hydrocarbons	−0.14	−0.14	−0.27	−0.23	−0.08	−0.20	0.00
Organic acids	−0.07	−0.07	−0.17	−0.06	0.00	0.01	−0.01
Fatty acids	−0.10	−0.10	−0.37*	−0.29	−0.24	−0.33	0.10
Sugars	−0.19	−0.19	−0.07	−0.16	0.35*	0.43*	−0.38*
Alcohols	−0.37*	−0.37*	−0.42*	−0.39*	0.23	0.13	−0.33

*Note:* Negative correlations are highlighted in shades of blue, positive ones in shades of red. Significant correlations are written in bold and marked with * for *p* ≤ 0.05 and ** for *p* ≤ 0.01.

## 4 Discussion

**FIGURE 5:**
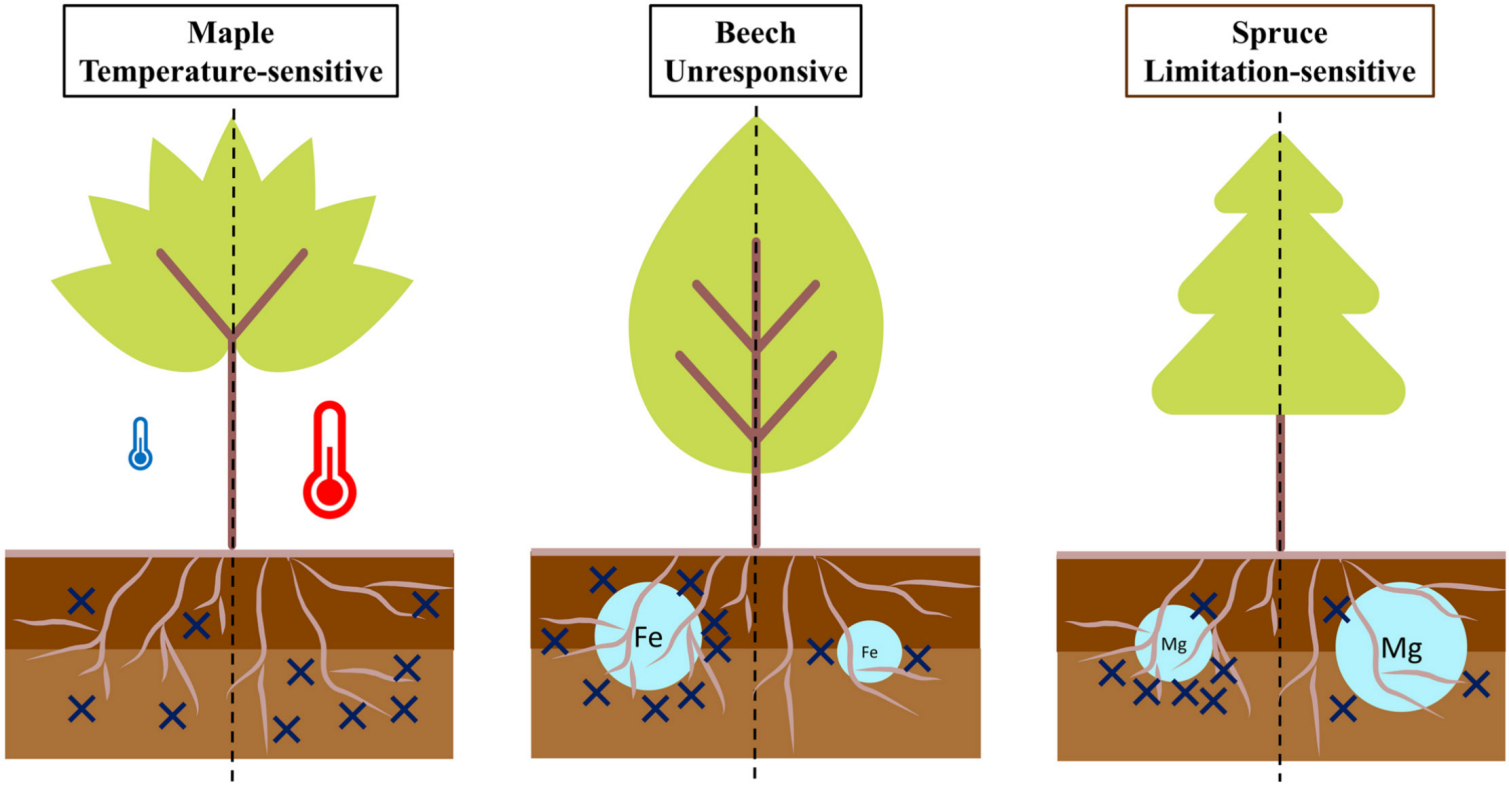
Root exudation strategies of three temperate tree species. Conceptual representation of root exudation patterns (blue crosses) in maple, beech and spruce in the forest floor (dark brown) and the mineral soil (light brown) in response to temperature (colored thermometers), season (flower: Late spring, apple: Late summer) and exchangeable soil cations (light blue circles).

The species‐specific root exudation patterns observed in this study suggest that environmental conditions, such as season, mean annual temperature, organic or mineral soil compartments, and nutrient availability are decisive factors for root exudation in temperate tree species. The three studied species showed distinct responses to these factors, stressing the importance of species‐specific data. In our study, the AM associated maple showed a generally higher exudation rate, and species responded differently to soil chemistry and soil compartment. Dynamics in root exudation were found even between roots of the same tree in different, but spatially close soil compartments, namely the forest floor and the mineral top soil (Figure 5). Some compound groups in the exudates, such as sugars and alcohols, reacted more strongly to soil chemistry than others, indicating not only quantitative but also composition‐specific alteration of root exudates in response to abiotic factors and site conditions.

### 4.1 Species‐Specific Root Exudation Patterns in Temperate Forests

The three studied tree species showed strong, site‐dependent differences in exudation patterns. Such species differences were also observed in other studies; however, results are not consistent.

For example, it has been stated that EM‐associated species like beech and spruce generally exude more C than AM‐associated species like maple (Brzostek et al. 2015; Tückmantel et al. 2017). This is reasoned by the capability of EM‐tree associations to produce extracellular enzymes, whereas AM‐tree associations often lack this feature (Read and Perez‐Moreno 2003). Supporting this, Yin et al. (2014) observed that beech exuded a significantly higher amount of C than maple on three out of four sampling dates. However, in the present study, we observed a significantly higher exudation of soluble compounds in maple than in spruce, contradicting the above‐mentioned findings. On average, maple individuals were smaller (and potentially younger) than the other species in this study, which could be a reason for an increased exudation. However, a correlation of root exudation to DBH in maple did not prove to be significant (Figure S11). Moreover, Tückmantel et al. (2017) brought forward that the reduced exudation in AM‐associated tree stands might be due to the higher bioavailability of nitrogen. This could explain the here‐observed patterns, since all sites in our study are beech‐dominated and therefore also dominated by beech litter. Moreover, the higher exudation in maple could be a result of AM associations acquiring more nutrients, while beech and spruce are known to recycle more nutrients within the tree (Lang et al. 2016). Stronger N recycling could also explain the tendency of beech and spruce to exude a lower share of N‐containing compounds compared to maple.

Besides, it has been argued that the decreased meristematic activity under P‐limiting conditions would lead to a decreased exudation at P‐poor sites (Canarini et al. 2019). However, other studies observed the inverse (Jones et al. 2004; Jones et al. 2009; Vives‐Peris et al. 2020) or no relationship at all in beech (Leuschner et al. 2022). Supporting the latter finding, we found beech and maple to exude similar amounts at P‐rich and P‐poor sites, while spruce exuded more at P‐poor sites (even though not significantly). However, it needs to be pointed out that these discrepancies could potentially also arise from varying methods of analysis. While it is rather new to apply a compounds‐specific approach to root exudates of temperate forest trees, many studies so far focused on total exuded carbon. It also becomes clear that the site conditions evaluated here could not explain the site effect on root exudation completely, which suggests complex interaction and additional factors affecting root exudation. To elucidate these, further research is needed.

### 4.2 Seasonal Dynamics in Root Exudation in Temperate Tree Species

Although there is a seasonal course of photosynthetic C assimilation in temperate forests (Carrara et al. 2004; Tyrrell et al. 2012), seasonal patterns in root exudation have not been found consistently (Phillips et al. 2008; Tückmantel et al. 2017) and other environmental factors such as drought might play a significant role.

In this study, however, we detected a difference between late spring and late summer, with generally higher exudation in late spring, when C assimilation is also higher. Similarly, higher exudation in summer compared to winter has been observed before (Chen et al. 2023). Further, in an early stage of the vegetation period, tree growth and therefore nutrient uptake are stronger compared to the late stage of the vegetation period, with higher uptake of P and N in spring (May) compared to autumn (September–October) in beech at three sites, including one studied here (Likulunga et al. 2022). Enhanced nutrient uptake could therefore explain the increased exudation in late spring. This is supported by the higher share of N‐containing compounds in late spring, which accelerates the biomass decomposition through a lower C/N ratio in the exudates (Meier et al. 2017). Adding to this, the net primary production of temperate forests is higher in May/June than in September, but respiration is still high in September (Carrara et al. 2004). Since a high net primary production is an indicator of growth, this suggests that growth, and hence nutrient demand, is higher in May/June. The high respiration in September indicates that the trees are still active but have a reduced nutrient demand. Next to the seasonal variation in nutrient uptake, there was a difference in drought index between the two seasons in 2023. The Standardised Precipitation Evaporation Index (SPEI; Vicente‐Serrano et al. 2010) was lower in late summer than in late spring at all sites. The SPEI (calculated based on the 6 months before sampling) changed from mild drought in late spring to moderate drought in late summer at the sites WAL, KAI, and KAN. At BBR, no indication of drought was evident in late spring, but a mild drought was shown in late summer by the index. The reduced C assimilation under drought (Bréda et al. 2006) could therefore also be a reason for the smaller exudation rates in late summer. This is supported by Li et al. (2025) and Dannenmann et al. (2009) showing reduced exudation rates under drought. The reduction in root exudation rates between late spring and late summer in this study could therefore be a consequence of the altered soil water content reflected by the SPEI values and not necessarily result from phenological causes, especially at the sites WAL, KAI, and KAN. Data from close by sites in the Upper Rhine valley (Haberstroh et al. 2025) and the Black Forest (Ettenheim, unpublished data, site described in Kinzinger et al. (2024)) with comparable precipitation dynamics in 2023 indicate that soil water contents were still rather high in late spring but dropped in June and remained at a low level until autumn. Thus, it is likely that the general soil moisture dynamics observed at these two sites were comparable to our study sites. This suggests that even though precipitation events occurred throughout the summer months until the end of the sampling period, it is likely that the soil water content was significantly decreased in late summer compared to late spring at our study sites (except BBR), which is supported by SPEI values. Reduced exudation rates in late summer could therefore reflect a resource‐saving mechanism of the trees under drought (Gargallo‐Garriga et al. 2018).

However, a range of studies found the opposite effect with increased exudation under drier conditions (Preece et al. 2018; Jakoby et al. 2020; Meier et al. 2020; Brunn et al. 2022; Leuschner et al. 2022), which argues against a soil water content induced change in exudation rate. These contradictory findings point to a significant knowledge gap and advocate for more drought‐related root exudation studies in temperate tree species (Ritter et al. 2025).

### 4.3 Root Exudation in the Forest Floor and the Mineral Soil

In this study, we found a comparatively low exudation in maple in the forest floor at the warm sites WAL and KAI, which have a thinner forest floor and therefore a lower share of organically bound nutrients, while the exudation at the colder P‐poor site KAN with a thick forest floor is higher in the forest floor compared to the mineral soil. The quantity of plant nutrients present in the organic or inorganic form was found to influence root exudation in various studies. Hereby, a positive relationship between organically bound N in the soil and root exudation C was found (Rohrbacher and St‐Arnaud 2016; Tückmantel et al. 2017; Vives‐Peris et al. 2020). Adding to this, it has been stated that the often observed increased exudation under N‐ and P‐limiting conditions (Jones et al. 2004; Jones et al. 2009; Vives‐Peris et al. 2020) only holds when N is present in the organic form (Meier et al. 2020). This could be a mechanism to increase carbon use efficiency by avoiding the production of compounds for priming effects when it is inefficient because microbial activity cannot increase the bioavailability of N if not present as organic N. This could explain our observed pattern in maple. Additionally, higher temperatures at WAL and KAI also most likely lead to a generally higher mineralisation rate, which could decrease the need of exudates for mineralisation. This pattern is not present in beech and spruce, which might be due to their above mentioned higher nutrient recycling capacity compared to maple, which belongs to the nutrient acquiring type (Lang et al. 2016). Those different nutrient economies are represented in the litter quality being low in nutrient recycling types, especially in spruce having a high C/N ratio in the litter (Vesterdal et al. 2008) and therefore in the forest floor. In contrast, the C/N ratio in the SOM in the mineral soil seems to be similar for the three species (Vesterdal et al. 2013). This could explain the increased exudation of spruce in the mineral soil, especially at the colder sites, where decomposition is slow. Even though many of the differences between soil compartments are not significant, they indicate that trees can adjust their root exudates on a very small scale, as between different soil compartments.

### 4.4 Root Exudation in Temperate Tree Species in Response to Soil Chemistry and Stand Characteristics

The capacity of dynamic adjustment is also represented in the significant relationships detected between root exudates and soil elements (Figure 4). We mainly identified significant relationships for root exudates versus exchangeable cations, representing the fact that the exchangeable form of elements is most important for temperate tree species. However, it remains unclear whether the abundance of exchangeable elements exerts an effect on the root exudates or vice versa. Malate, pyruvate, and citrate have been identified to be helpful to withstand Al toxicity (Pantigoso et al. 2021). Previous studies have already found indications of Al^3+^ complexation through root exudates to reduce phytotoxic effects (Heim et al. 2001; Collignon et al. 2012). This could explain the positive relationship between the exudation of hydrocarbons and exchangeable non‐base cations in the soil in beech and spruce in our study. In our case, other N hydrocarbons (for beech) and sugars (for spruce) were involved, which could be a species‐specific reaction. The same could hold for beech, which also increased exudation with increasing non‐base cation concentration. In beech, however, Fe^2+^ seemed to have a stronger effect than Al^3+^, which could also be a species‐specific reaction.

The relationship between base cations and root exudation is less studied. Interestingly, beech and spruce showed inverse patterns in this regard. The increased rate of total exudation, amino acid, and alcohol exudation in spruce under decreased exchangeable base cation concentrations in the soil could increase the mobility of those elements by desorbing them from, for example, clay minerals (alcohols and amino acids) and decreasing the C/N ratio in the soil (amino acids) which accelerates the SOM degradation through microorganisms (Meier et al. 2017). The latter mechanism is especially relevant for spruce because of the high C/N ratio in its litter (Vesterdal et al. 2013). In contrast, N‐containing compounds in the exudates of beech were not related to the abundance of exchangeable base cations. Instead, we found the exudation of organic acids, fatty acids, and sugars to correlate positively with the exchangeable base cation Mg^2+^. The observed pattern could be a hint for higher Mg^2+^ concentrations being a consequence and not a cause of exudation by beech. This mechanism has been observed for organic acid exudation in *Cryptomeria japonica* (Ohta and Hiura 2016). However, this effect could not be seen in the other species and needs more research to be fully understood. Further compound‐targeted studies could reveal the specific role of beech in this case. Nonetheless, it is a further indicator of pronounced species differences and shows that not only the rate of exudation, but also its composition is dynamically adjusted to abiotic factors.

Regarding the possible influence of stand characteristics, tree species mixing has been reported to impact the composition of root exudates, yet no differences in total exuded carbon were found (He et al. 2025). Specifically, the exudation of amino acids and peptides tended to be higher in pure stands compared to mixed stands (He et al. 2025). This is in line with the lower rate of amino acid exudation in our study at WAL, which has a higher share of species other than beech compared to the other study sites. However, this effect was not significant. Also, it remains unclear whether this difference was caused by a different tree species composition or other site conditions. To our knowledge, the effect of stand density on root exudation has not been a focus of research, and we could not find a clear pattern in our data. However, a higher stand basal area increases tree competition for nutrients (Barbosa et al. 2023). Therefore, it seems likely that stand density could also affect root exudation. Future studies should investigate root exudation at sites with differing basal areas but otherwise similar conditions to close this knowledge gap.

## 5 Conclusion

While spruce showed a tendency to exude more in the mineral soil and reacted sensitively to nutrient availability with increased exudation under limiting conditions of P and base cations (limitation‐sensitive exudation), maple seems to respond more strongly to temperatures with higher exudation rates at warm sites, especially in the mineral soil (temperature‐sensitive exudation). The exudation of beech was related to the concentration of non‐base cations, with higher concentrations inducing higher exudation, which might be a protection mechanism against phytotoxicity, but was not related to soil compartment nor temperature. However, beech showed the highest decrease in exudation between late spring and late summer (exudation sensitive to season; Figure 5).

This strongly indicates species‐specific adaptations and dynamics of root exudation to season, soil compartment, and soil chemistry. Interestingly, roots from the same trees showed differences in exudation in the two soil compartments. This suggests that an active response in root exudation to specific conditions in the forest floor, with lower risk of Al toxicity and a higher share of organically bound nutrients, is possible. The dynamics in root exudation were also reflected in the seasonal patterns and adjustments to the abundance of exchangeable soil cations.

The results of this study show that root exudation reacts dynamically to environmental parameters. Therefore, gaining knowledge about root exudates, especially in field environments, is particularly challenging. Analyzing the composition next to the rate of exudates allows more detailed insights into root exudation patterns, drivers, and functions. Compound analyses might give a clue to so far often contrasting findings. For instance, in this study, a strong response of the sugar exudation in spruce to the concentration of non‐base cations was found, which was not significantly reflected in total exudation rates.

## Author Contributions

C.W., J.K., M.W., and S.H. designed the study. M.W. conducted the field and lab work. J.K. assisted during sample processing and analysis. M.W. drafted the manuscript with major contributions from S.H. C.W. and S.H. helped with data analysis and interpretation. J.N., J.P., and F.L. contributed site and soil chemistry data, and T.H.D. contributed forest stand structure data. All authors critically reviewed the manuscript and contributed substantially.

## Funding

This research was funded by the DFG (project no.: WE2681/13‐1) in the frame of the Research Unit 5315 ‘Forest Floor: Functioning, Dynamics and Vulnerability in a Changing World’.

## Conflicts of Interest

The authors declare no conflicts of interest.

## Supporting information


**Data S1:** Supporting Information.

## Data Availability

The data that support the findings of this study are available from the corresponding author upon reasonable request.
