# Supplementary material for: Spatio‐Temporal Plasticity of Root Exudation in Three Temperate Tree Species: Effects of Season, Site, and Soil Characteristics
Source: Physiol Plant. 2025 Dec 17;177(6):e70681. doi: 10.1111/ppl.70681 (PMC12710586; doi:10.1111/ppl.70681)
Supplement: Supplementary file 1 — Data S1: Supporting Information. [file PPL-177-e70681-s001.docx]

Supplementary data

Spatio-temporal plasticity of root exudation in three temperate tree species: effects of season, site and soil characteristics

Melissa Wannenmacher^1^; Simon Haberstroh^1^; Jürgen Kreuzwieser^1^; Trung Hieu Doan^2^; Jörg Niederberger^3^; Jörg Prietzel^4^, Friederike Lang^3^; Christiane Werner^1^

^1^Ecosystem Physiology, Faculty of Environment and Natural Resources, University of Freiburg, Germany

^2^Silviculture, Faculty of Environment and Natural Resources, University of Freiburg, Germany
^3^Soil Ecology, Faculty of Environment and Natural Resources, University of Freiburg, Germany
^4^Renaturation Ecology, TUM School of Life Sciences, Technical University of Munich, Germany

Corresponding author: melissa.wannenmacher@cep.uni-freiburg.de


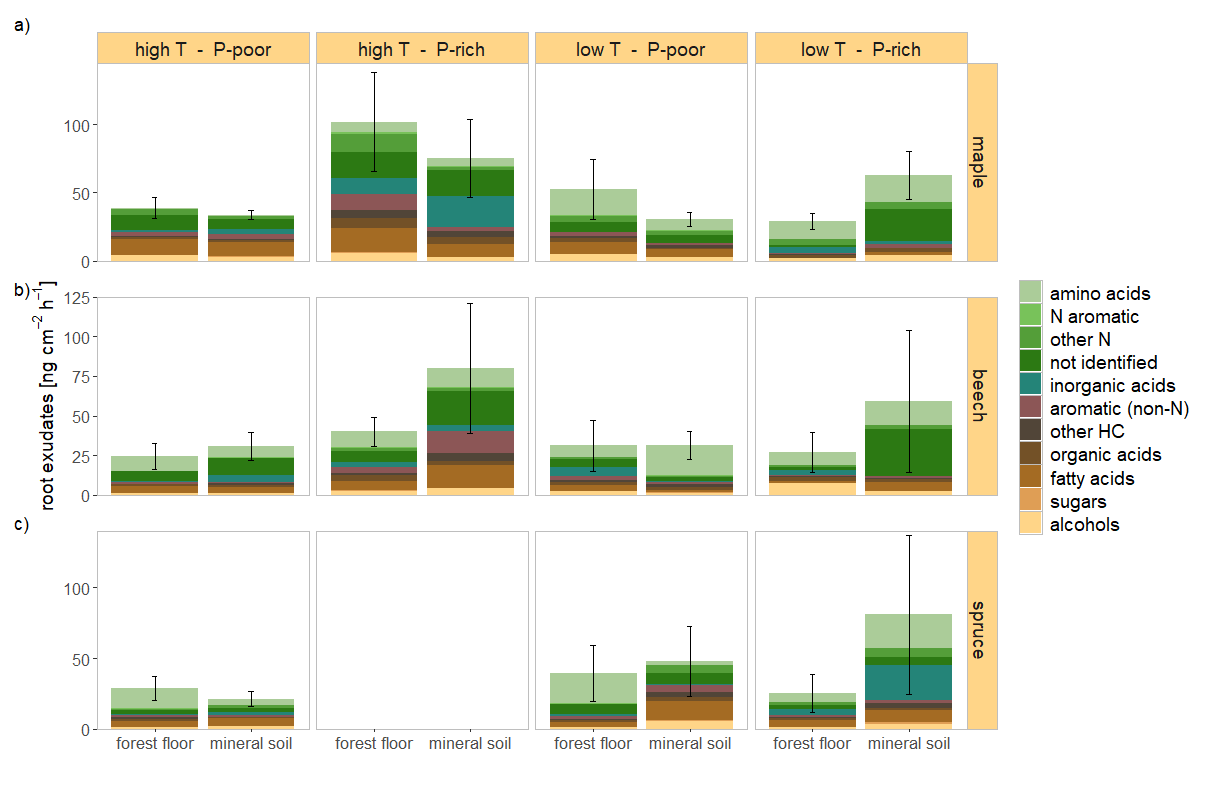


**Figure S1**: Root exudation in late summer in the forest floor and the mineral soil for maple in a), beech in b) and spruce in c) at the four study sites. Colours code compound groups within the exudates. Note the different scales for different species.


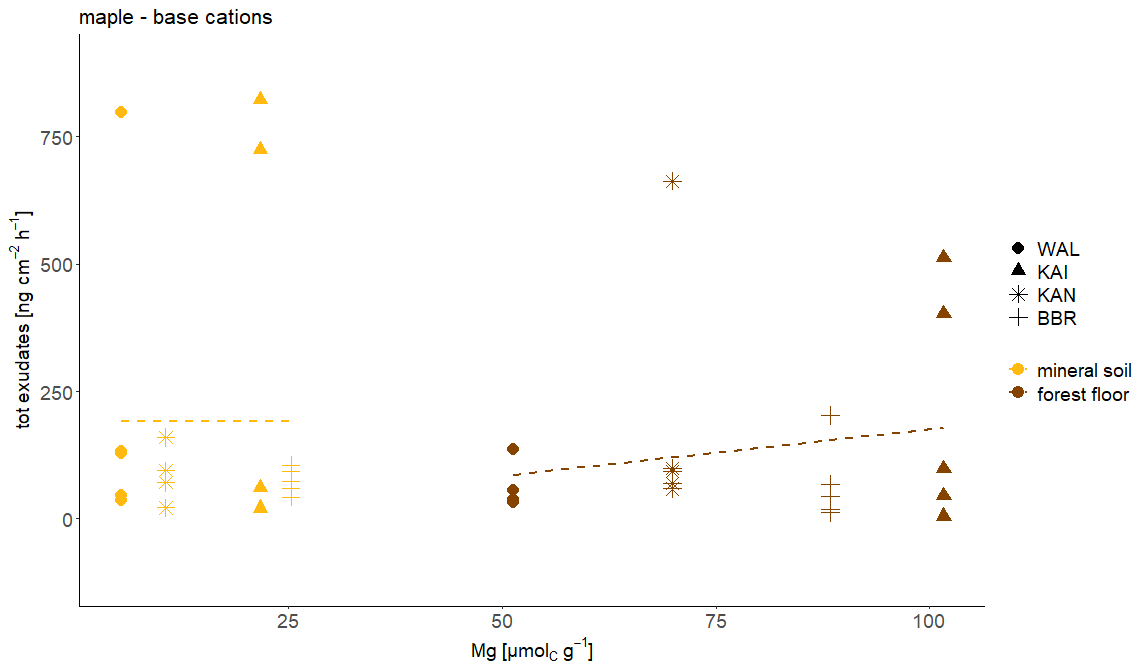


**Figure S2**: Total exudates in relation to exchangeable Mg concentration for maple. Shapes indicate the four different sites and the colours the horizon. Regression lines are illustrated by generalized linear smoothing.


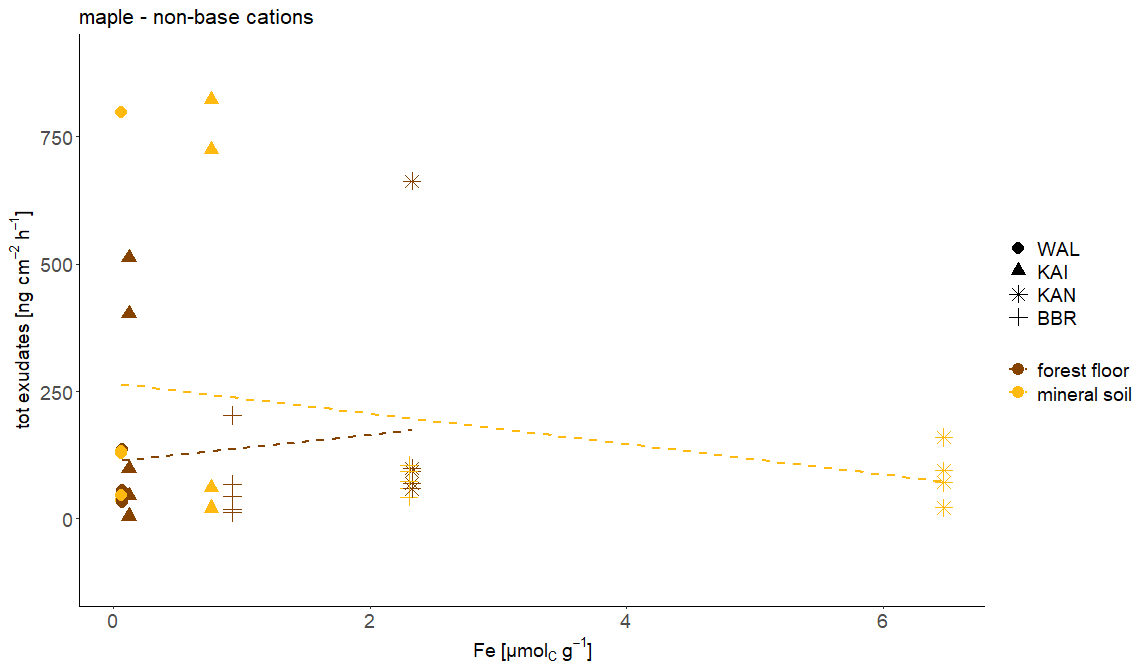


**Figure S3**: Total exudates in relation to exchangeable Fe concentration for maple. Shapes indicate the four different sites and the colours the horizon. Regression lines are illustrated by generalized linear smoothing.


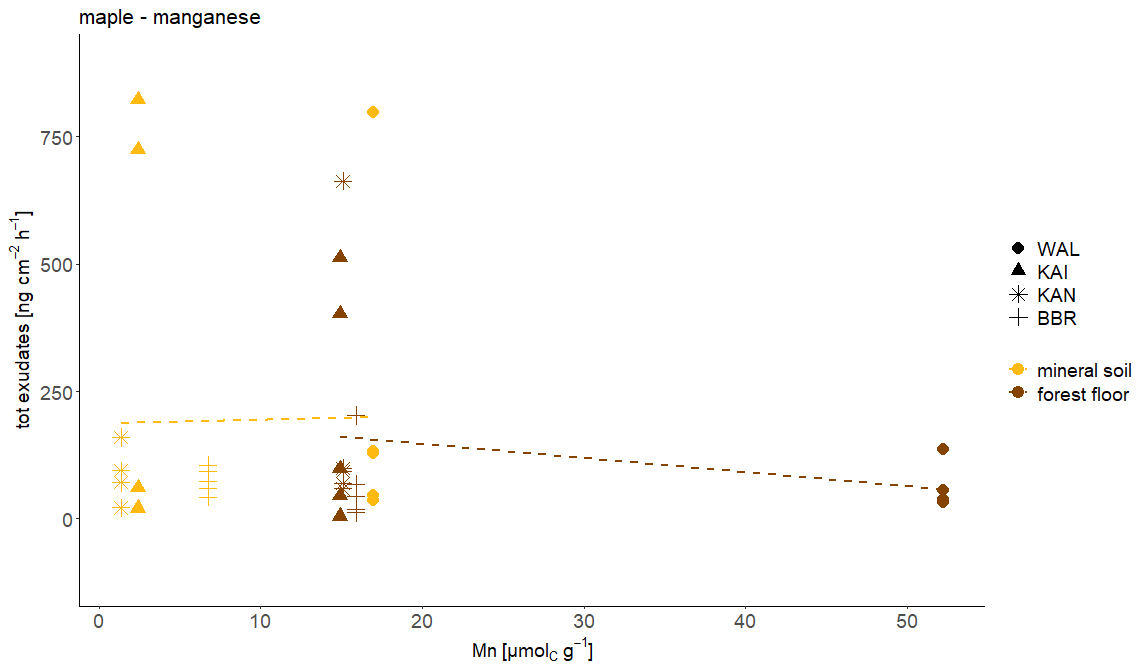


**Figure S4**: Total exudates in relation to exchangeable Mn concentration for maple. Shapes indicate the four different sites and the colours the horizon. Regression lines are illustrated by generalized linear smoothing.


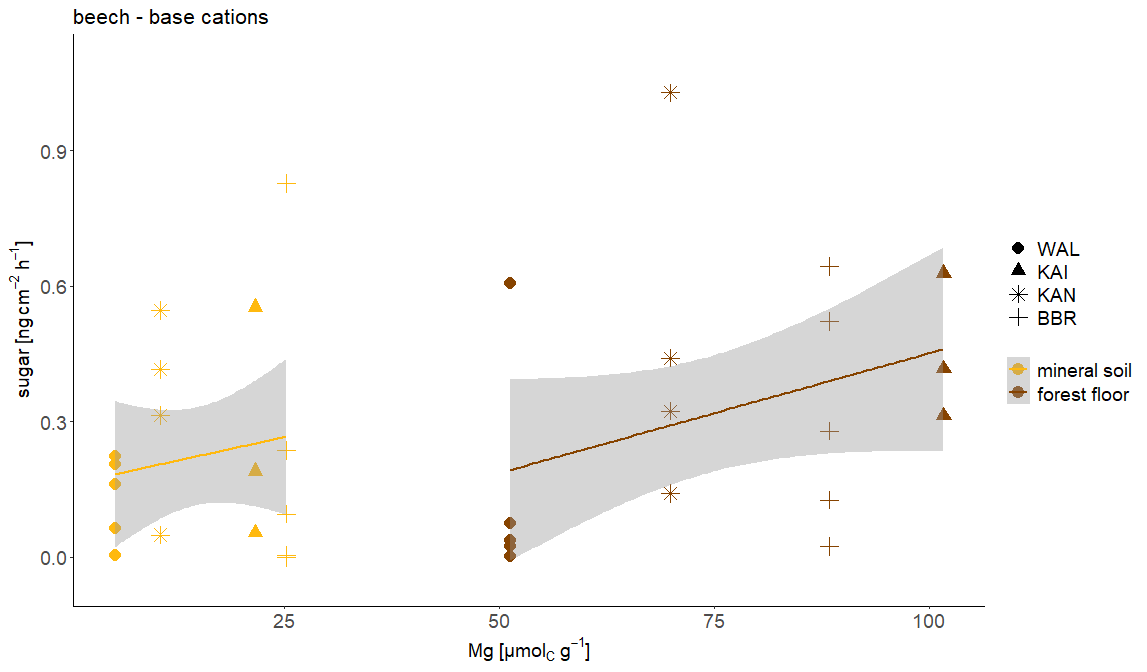


**Figure S5**: Sugar exudation in relation to exchangeable Mg concentration for beech. Shapes indicate the four different sites and the colours the horizon. Regression lines are illustrated by generalized linear smoothing and the shaded areas indicate the 95% confidence interval.


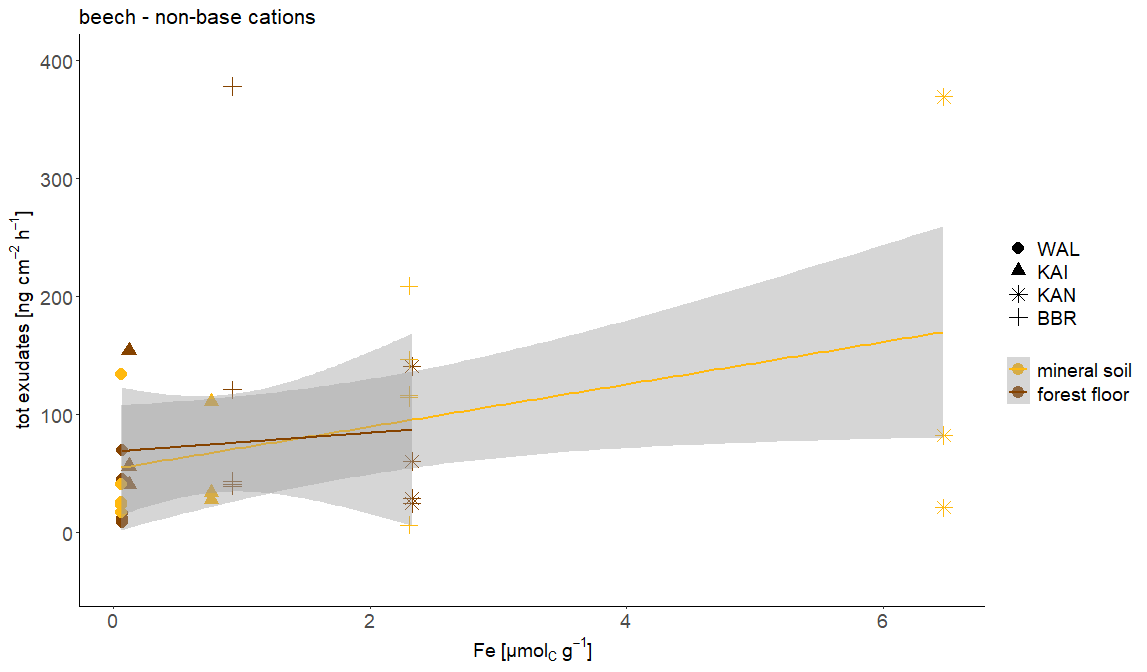


**Figure S6**: Total exudates in relation to exchangeable Fe concentration for beech. Shapes indicate the four different sites and the colours the horizon. Regression lines are illustrated by generalized linear smoothing and the shaded areas indicate the 95% confidence interval.


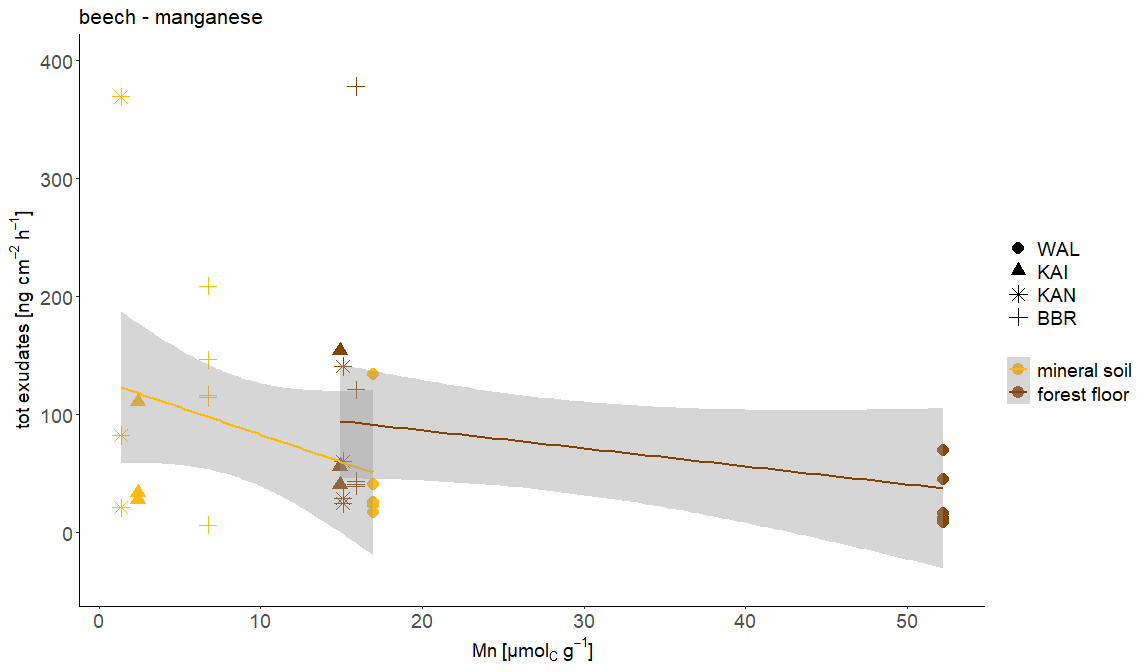


**Figure S7**: Total exudates in relation to exchangeable Mn concentration for beech. Shapes indicate the four different sites and the colours the horizon. Regression lines are illustrated by generalized linear smoothing and the shaded areas indicate the 95% confidence interval.


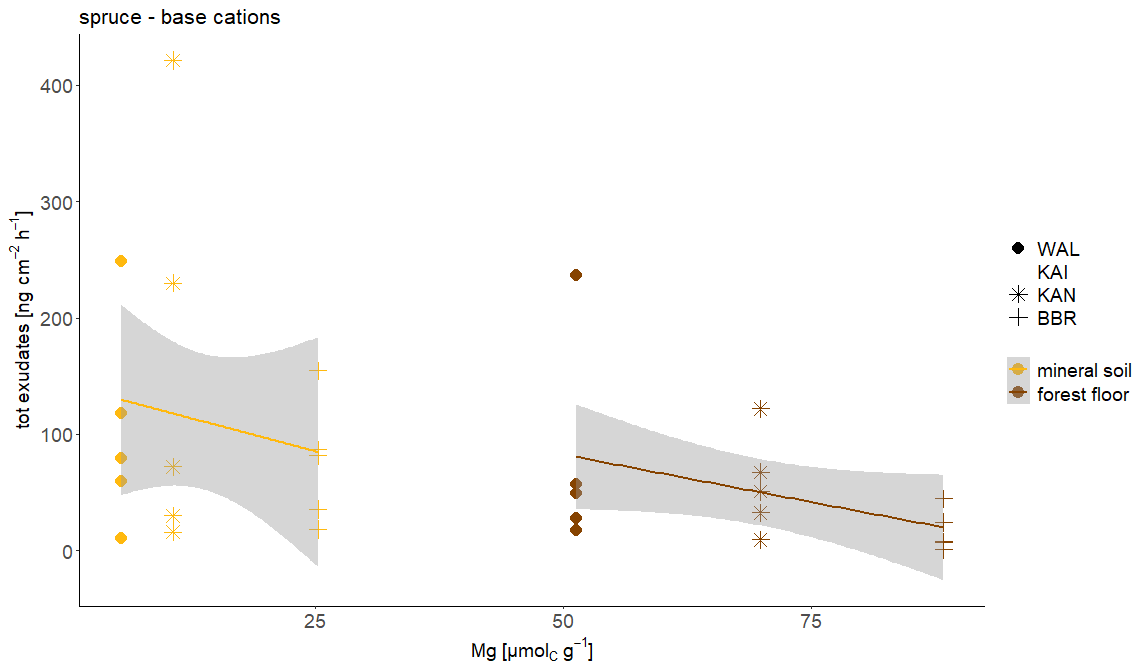


**Figure S8**: Total exudates in relation to exchangeable Mg concentration for spruce. Shapes indicate the four different sites and the colours the horizon. Regression lines are illustrated by generalized linear smoothing and the shaded areas indicate the 95% confidence interval.


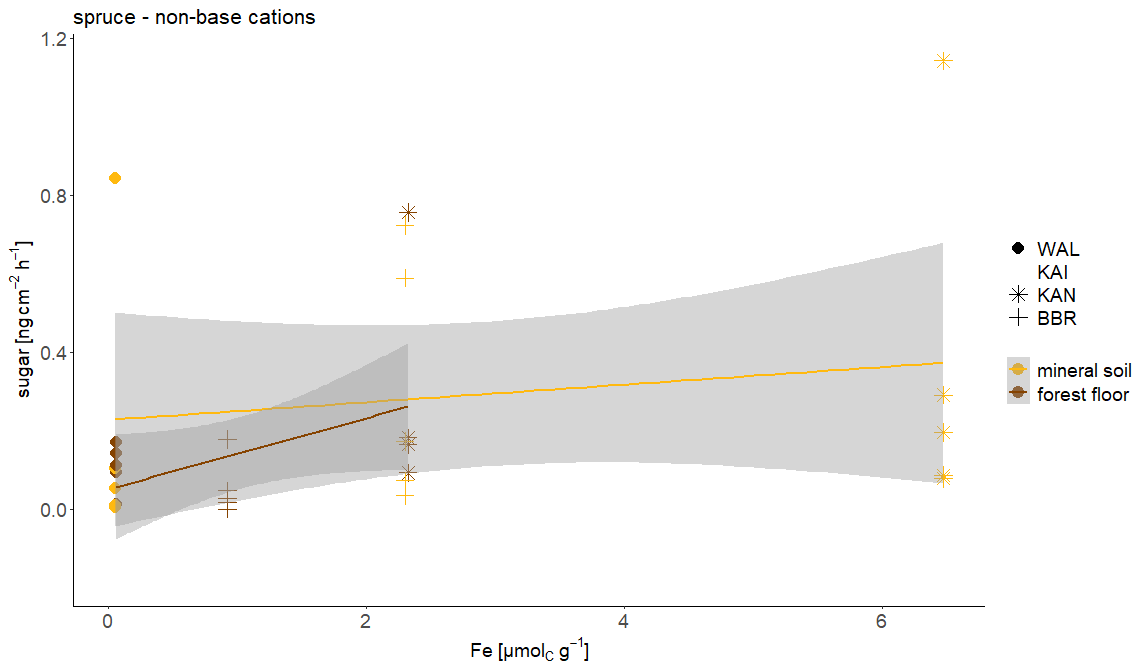


**Figure S9**: Sugar exudation in relation to exchangeable Fe concentration for spruce. Shapes indicate the four different sites and the colours the horizon. Regression lines are illustrated by generalized linear smoothing and the shaded areas indicate the 95% confidence interval.


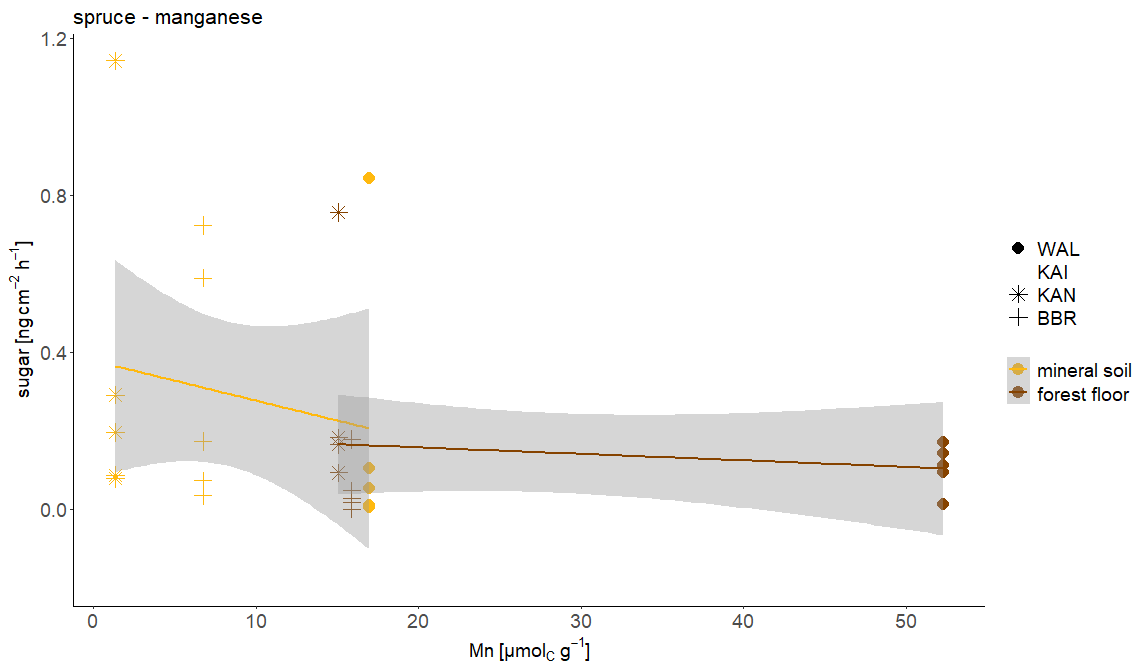


**Figure S10**: Sugar exudation in relation to exchangeable Mn concentration for spruce. Shapes indicate the four different sites and the colours the horizon. Regression lines are illustrated by generalized linear smoothing and the shaded areas indicate the 95% confidence interval.


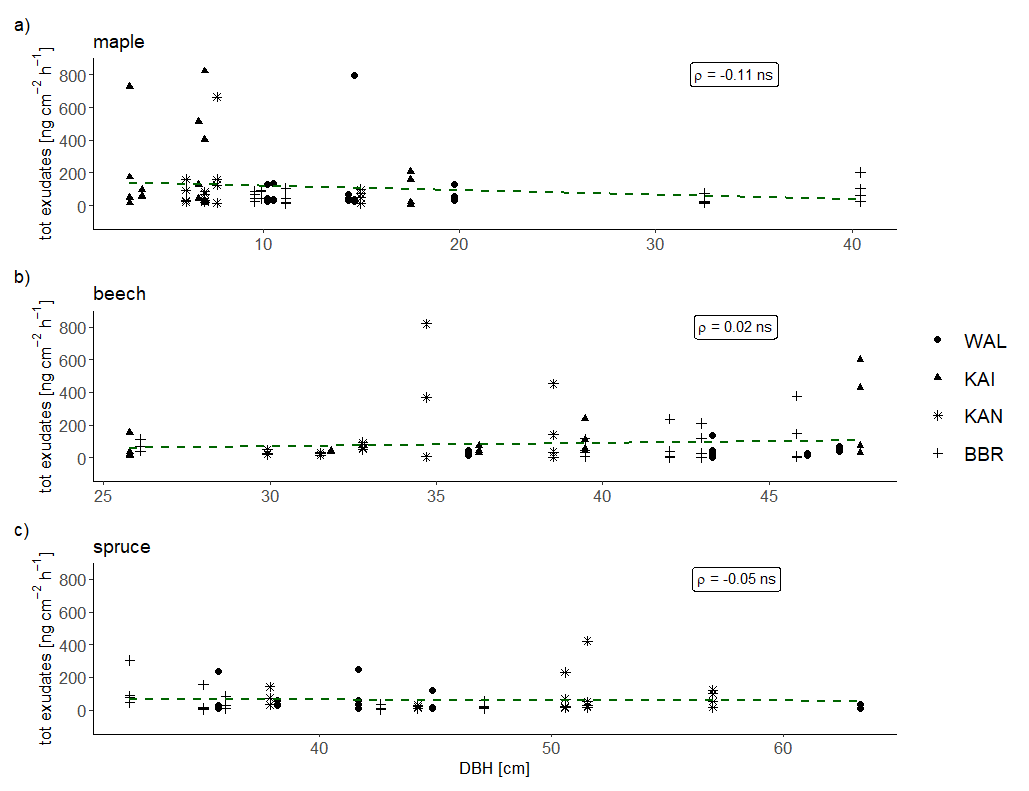


**Figure S11**: Total exudation in relation to diameter at breast height (DBH) for maple in a), for beech in b) and spruce in c). ρ indicates the Spearman’s rank correlation coefficient and “ns” indicates that correlations were non-significant.

**Table S1**: Compounds detected in GC-MS analysis with retention time (RT), match factor, CAS number, mass-to-charge ratio (MZ), the standard used for quantification (also see Table S2) and the assigned compound group.

| **Name** | **RT** | **Match Factor** | **CAS#** | **MZ** | **standard for quantification** | **compound group** |
| --- | --- | --- | --- | --- | --- | --- |
| 1,2,3-Propanetriol, 1-(4-hydroxy-3-methoxyphenyl)- (4TMS) | 29.21 | 79 |  | 297.0 | alcohol | alcohol |
| 1-Pyrroline-2-carboxylate (1TMS) | 10.80 | 81 |  | 170.0 | other_N | other_N |
| 4-Hydroxyphenyl-beta-glucopyranoside (5TMS) | 38.80 | 67 |  | 254.0 | arom | arom |
| Alanine (3TMS) | 14.82 | 74 |  | 188.0 | amino_acid | amino_acid |
| Alanine, beta- (1TMS) | 4.67 | 69 | 5269-40-9 | 117.0 | amino_acid | amino_acid |
| Arabitol (5TMS) | 23.67 | 68 | 14199-73-6 | 217.0 | alcohol | alcohol |
| Benzoic acid, (1TMS) | 10.24 | 71 |  | 179.0 | arom | arom |
| Benzylalcohol (1TMS) | 8.00 | 82 | 14642-79-6 | 165.0 | alcohol | alcohol |
| beta-D-Fructofuranosyl-(2,1)-beta-D-Fructofuranose (1MEOX) (8TMS) BP | 23.10 | 72 |  | 217.0 | sugar | sugar |
| Boric-acid_3TMS | 4.71 | 75 |  | 221.0 | boric acid | other_acid |
| Butane, 1,2,4-trihydroxy- (3TMS) | 22.71 | 66 | 33581-75-8 | 103.0 | other_HC | other_HC |
| Butanoic acid, 3-hydroxy- (2TMS) | 9.78 | 73 | 55133-94-3 | 191.0 | org_acid | org_acid |
| Butanoic acid, 4-amino- (3TMS) | 18.93 | 85 | 39508-23-1 | 174.0 | amino_acid | amino_acid |
| Butyro-1,4-lactam (1TMS) | 8.87 | 63 | 14468-90-7 | 142.0 | other_N | other_N |
| Carbodiimide (2TMS) | 4.31 | 83 | 1000-70-0 | 171.0 | other_N | other_N |
| Cysteamine (3TMS) | 4.12 | 76 |  | 174.0 | other_N | other_N |
| D204282 | 23.25 | 61 |  | 147.0 | other | other |
| Decanoic acid (1TMS) | 15.54 | 66 | 55494-15-0 | 229.0 | fatty_acid | fatty_acid |
| Diethanolamine (2TMS) | 13.96 | 68 |  | 131.0 | other_N | other_N |
| Dihydroxyphenylalanine_3TMS | 27.64 | 71 |  | 267.0 | amino_acid | amino_acid |
| Dodecane | 23.15 | 77 | 112-40-3 | 85.0 | other_HC | other_HC |
| Ethanolamine (3TMS) | 4.14 | 67 | 5630-81-9 | 174.0 | other_N | other_N |
| Fructose (1MEOX) (5TMS) MP | 25.46 | 76 |  | 307.0 | fructose | sugar |
| Galactitol (6TMS) | 25.00 | 65 |  | 217.0 | alcohol | alcohol |
| Galactose (1MEOX) (5TMS) MP | 25.96 | 79 |  | 319.0 | galactose | sugar |
| Glucopyranose [-H20] (4TMS) | 24.43 | 68 |  | 217.0 | sugar | sugar |
| Glycerol (3TMS) | 11.24 | 80 | 06/10/6787 | 147.0 | glycerol | alcohol |
| Glycine, N,N-dimethyl- (1TMS) | 4.19 | 72 |  | 160.0 | N,N-dimethyl-glycine | amino_acid |
| Heneicosan-1-ol, n- (1TMS) | 11.68 | 62 |  | 369.0 | alcohol | alcohol |
| Hexadecanoic acid (1TMS) | 28.06 | 76 |  | 313.0 | fatty_acid | fatty_acid |
| Hexanoic acid (1TMS) | 6.23 | 73 | 14246-15-2 | 173.0 | org_acid | org_acid |
| Hydrogen sulfide (2TMS) | 5.36 | 91 | 3385-94-2 | 163.0 | other | other |
| Hydroxylamine (3TMS) | 7.20 | 85 | 21023-20-1 | 249.0 | other_N | other_N |
| Inositol, myo- (6TMS) | 28.31 | 84 |  | 318.0 | alcohol | alcohol |
| Isoleucine (2TMS) | 13.20 | 70 | 7483-92-3 | 158.0 | amino_acid | amino_acid |
| Lactic acid (2TMS) | 6.08 | 65 | 17596-96-2 | 147.0 | lactic acid | org_acid |
| Lyxose (1MEOX) (4TMS) BP | 22.20 | 69 |  | 307.0 | sugar | sugar |
| Mannitol (6TMS) | 27.85 | 88 |  | 319.0 | alcohol | alcohol |
| NA | 11.60 | 72 |  | 174.0 | other | other |
| NA114002 (classified unknown) | 7.77 | 75 |  | 147.0 | other | other |
| NA192001 (classified unknown) | 26.57 | 72 |  | 319.0 | other | other |
| NA199017 | 26.65 | 64 |  | 110.0 | other | other |
| Nicotinic acid (1TMS) | 13.02 | 80 | 25436-37-7 | 180.0 | arom_N | arom_N |
| Nonanoic acid (1TMS) | 13.14 | 67 | 82326-11-2 | 215.0 | fatty_acid | fatty_acid |
| Octadecanoic acid (1TMS) | 31.58 | 64 |  | 341.0 | fatty_acid | fatty_acid |
| Octan-1-ol, n- (1TMS) | 6.92 | 81 | 14246-16-3 | 187.0 | alcohol | alcohol |
| Octanoic acid, n- (1TMS) | 10.68 | 72 | 55494-06-9 | 201.0 | fatty_acid | fatty_acid |
| Ononitol (5TMS) | 27.80 | 79 |  | 305.0 | alcohol | alcohol |
| O-Toluic-acid_1TMS | 13.21 | 65 |  | 193.0 | arom | arom |
| Palmitic-acid-amide | 26.51 | 63 |  | 194.0 | other_N | other_N |
| Phenol (1TMS) | 7.19 | 69 |  | 151.0 | arom | arom |
| Pinitol, D- (5TMS) | 25.85 | 93 |  | 260.0 | alcohol | alcohol |
| Prolyl-glycine (2TMS) | 10.47 | 63 |  | 142.0 | amino_acid | amino_acid |
| Propane-1,2-diol (2TMS) | 12.55 | 71 | 17887-27-3 | 117.0 | alcohol | alcohol |
| Propane-1,3-diol (2TMS) | 5.96 | 62 | 17887-80-8 | 147.0 | propane-1,3-diol | alcohol |
| Propane-1,3-diol, 2-amino-2-methyl- (2TMS) | 15.65 | 66 |  | 130.0 | other_N | other_N |
| Putrescine (4TMS) | 23.63 | 65 | 39772-63-9 | 174.0 | other_N | other_N |
| Pyridine, 2-hydroxy- (1TMS) | 6.83 | 78 |  | 152.0 | arom_N | arom_N |
| Quinic acid (5TMS) | 26.41 | 61 |  | 345.0 | org_acid | org_acid |
| similar to Ditertbutylphenol (1TMS) | 17.69 | 75 |  | 263.0 | other | other |
| similar to Inositol (6TMS) | 29.50 | 62 |  | 305.0 | other | other |
| Sorbitol, 1,4:3,6-dianhydro- (2TMS) | 16.96 | 77 |  | 275.0 | alcohol | alcohol |
| Sorbose (1MEOX) (5TMS) BP | 22.33 | 66 |  | 217.0 | sugar | sugar |
| Threitol (4TMS) | 18.71 | 69 | 32381-52-5 | 217.0 | alcohol | alcohol |
| Triethanolamine (3TMS) | 19.84 | 67 | 20836-42-4 | 262.0 | triethanolamine | other_N |
| Tryptophan, 5-hydroxy- (3TMS) MP | 16.88 | 61 |  | 218.0 | arom_N | arom_N |
| Unknown#bth-pae-001 | 5.75 | 80 |  | 214.0 | other | other |
| Unknown#bth-pae-013 | 10.16 | 83 |  | 281.0 | other | other |
| Unknown#bth-pae-039 | 24.40 | 66 |  | 217.0 | other | other |
| Uracil (2TMS) | 14.31 | 65 | 10457-14-4 | 241.0 | other_N | other_N |
| Urea (2TMS) | 10.09 | 67 |  | 171.0 | other_N | other_N |
| Xylose (1MEOX) (4TMS) MP | 25.69 | 66 | 56196-07-7 | 307.0 | sugar | sugar |

**Table S2**: List of authentic standards and their conversion factors. Compounds without authentic standard were converted based on a calculated conversion factor for the respective compound group.

| **measured conversion factors** | |  |
| --- | --- | --- |
| **standard** | **conversion factor [g/peak area unit]** |  |
| aspartic acid | 4.40E-12 |  |
| boric acid | 6.13E-12 |  |
| citric acid | 1.86E-11 |  |
| cysteine | 8.63E-13 |  |
| fructose | 6.16E-12 |  |
| galactose | 3.19E-12 |  |
| glutamic acid | 4.07E-11 |  |
| glycerol | 1.40E-11 |  |
| lactic acid | 1.22E-11 |  |
| malic acid | 1.11E-11 |  |
| N,N-dimethyl-glycine | 5.12E-12 |  |
| phenylalanine | 1.80E-12 |  |
| propane-1,3-diol | 2.26E-11 |  |
| pyruvic acid | 1.12E-10 |  |
| sucrose | 1.16E-12 |  |
| triethanolamine | 6.44E-12 |  |
| uric acid | 3.06E-10 |  |
|  |  |  |
| **calculated conversion factors** | |  |
| **standard** | **conversion factor [g/peak area unit]** | **mean conversion factor from:** |
| amino_acid | 1.06E-11 | aspartic acid, cysteine, glutamic acid, N,N-dimethyl-glycine, phenylalanine |
| arom_N | 9.88E-12 | aspartic acid, cysteine, glutamic acid, N,N-dimethyl-glycine, phenylalanine, triethanolamine |
| arom | 9.21E-11 | citric acid, lactic acid, malic acid, pyruvic acid, uric acid |
| other_N | 6.44E-12 | triethanolamine |
| other_HC | 5.07E-11 | citric acid, fructose, galactose, glycerol, lactic acid, malic acid, propane-1,3-diol, pyruvic acid, sucrose, uric acid |
| other | 3.37E-11 | all |
| alcohol | 1.83E-11 | glycerol, propane-1,3-diol |
| org_acid | 9.21E-11 | citric acid, lactic acid, malic acid, pyruvic acid, uric acid |
| fatty_acid | 9.21E-11 | citric acid, lactic acid, malic acid, pyruvic acid, uric acid |
| sugar | 3.50E-12 | fructose, galactose, sucrose |
| other_acid | 5.14E-11 | aspartic acid, boric acid, citric acid, cysteine, glutamic acid, lactic acid, malic acid, phenylalanine, pyruvic acid, uric acid |
